# *Inhba, Homer1* and *Bdnf* are major targets of transcriptomic dysregulation by neurodegenerative disease-associated excitotoxic NMDA receptor signaling

**DOI:** 10.1038/s42003-025-09074-9

**Published:** 2025-12-03

**Authors:** Kristin Oberländer, Priit Pruunsild, Philipp Koch, Jing Yan, Karol Szafranski, Hilmar Bading

**Affiliations:** 1 Department of Neurobiology, Heidelberg University, Heidelberg, Germany; 2 CF Life Science Computing, Leibniz Institute on Aging – Fritz Lipmann Institute (FLI) e.V., Jena, Germany; 3 Network Aging Research, Heidelberg University, Heidelberg, Germany; 4 Present Address: Translational Dementia Research, German Center for Neurodegenerative Diseases, Bonn, Germany; 5 Present Address: FundaMental Pharma GmbH, Heidelberg, Germany

**Keywords:** Molecular neuroscience, Diseases of the nervous system

## Abstract

Synaptic activity-regulated gene expression supports neuroprotection, plasticity, and memory. The transcription factor CREB is central to these processes. It is activated by synaptic NMDA receptors but inactivated by excitotoxic extrasynaptic NMDAR (esNMDAR) signaling. Using primary hippocampal neurons, we modeled neurodegeneration and found that esNMDAR activation, which causes CREB shut-off and inactivation of the ERK/MAPK-ELK1/SRF pathway, extensively distorted control of synaptic activity over transcription. This resulted in the suppression of key neuroprotective genes, in particular *Inhba and Bdnf*, but also of genes involved in synaptic function (*Homer1*, *Btg2*, *Mir132*, *Mir212*) and transcription factor genes (*Atf3*, *Egr1*, *Fos*, *Npas4*). In a Huntington’s disease (HD) mouse model, treatment with memantine or targeting the NMDAR/TRPM4 complex with FP802 restored gene expression, notably *Inhba*, *Homer1* and *Bdnf*, and attenuated the decrease of the HD disease marker *Ppp1r1b* (DARPP-32). These findings identify esNMDAR-driven transcriptional dysregulation as a key pathomechanism in neurodegenerative disease, supporting inhibition of esNMDAR-signaling as a promising therapeutic approach.

## Abstract (web summary)

Activation of extrasynaptic NMDA receptors (esNMDAR) suppresses key neuroprotective and synaptic functionrelated genes. Targeting esNMDAR signaling in a Huntington´s disease mouse model can partially restore this transcriptional dysregulation.

## Introduction

Synaptic N-methyl-D-aspartate (NMDA) receptor (sNMDAR) signaling evokes a genomic transcriptional response that promotes neuroprotection and plasticity^1–4^. If NMDARs are stimulated extrasynaptically, however, they can override the positive effects of synaptic activity and impair neuronal health^5^. Specifically, extrasynaptic NMDAR (esNMDAR) activation causes transcriptional dysregulation, mitochondrial damage, and structural disintegration that constitute common pathomechanisms of excitotoxicity in various neurodegenerative conditions^6–8^.

A key mechanism that can at least in part explain the disruptive gene regulatory changes upon pathologically elevated esNMDAR signaling has been termed “CREB shut-off,” which refers to the inactivation by dephosphorylation of the transcription factor cAMP response element binding protein (CREB)^5,9^. CREB is critical for neuronal activity-responsive gene expression in neurons^3,10,11^ and controls memory formation^12,13^, neuron survival^14^, and activity-dependent acquired neuroprotection^1,2^, denoting the processes that can be directly affected by transcriptional dysregulation due to CREB shut-off. The target genes of both neuroprotective sNMDAR signaling and CREB largely overlap and have been comprehensively studied^1,2,11,15^. However, the transcriptional alterations caused by interference of esNMDAR signaling with synaptic signaling are poorly understood, as the only notable, but potentially functionally important, example of the CREB shut-off mechanism is the suppression of induction of the neurotrophin-encoding *Bdnf* gene^5^.

Toxic esNMDAR signaling has been implicated in the etiologies of different neurodegenerative diseases, including Huntington’s disease (HD)^16,17^, Alzheimer’s disease (AD)^18–20^, and amyotrophic lateral sclerosis (ALS)^21–23^. In these, but also in other neuropathological conditions where there is damage to nervous tissue, glutamate uptake is often impaired, causing spillover of synaptic glutamate to the extrasynaptic space and resulting in esNMDAR stimulation in the presence of synaptic signals^7,24^. In HD, the excitotoxic component is further enhanced by increased expression of NMDARs at extrasynaptic sites^17,25^. We hypothesized that, similarly to *Bdnf* suppression, the regulation of numerous other synaptic activity-responsive genes is likely to be affected by disease-associated esNMDAR activation. We therefore sought in this study to resolve the transcriptomic changes in conditions that mimic the typical neurodegenerative states where excitotoxic esNMDAR signaling perturbs responses evoked by synaptic activity. We employed a protocol using mouse primary hippocampal neurons that allowed us to identify the pool of activity-driven genes that is subject to dysregulation by toxic signaling induced by the stimulation of esNMDARs. Moreover, we provide evidence that the transcriptional dysregulation that results—at least in part—from esNMDAR activity in HD model mice can be rescued with the NMDAR open-channel blocker, memantine^26^, or the small molecule TwinF interface inhibitor, FP802, that abolishes excitotoxic signaling by disruption of the interaction between esNMDARs and transient receptor potential cation channel subfamily M member 4 (TRPM4) channels^8,23,27,28^.

## Results

### Rationale and study design

**Fig. 1:**
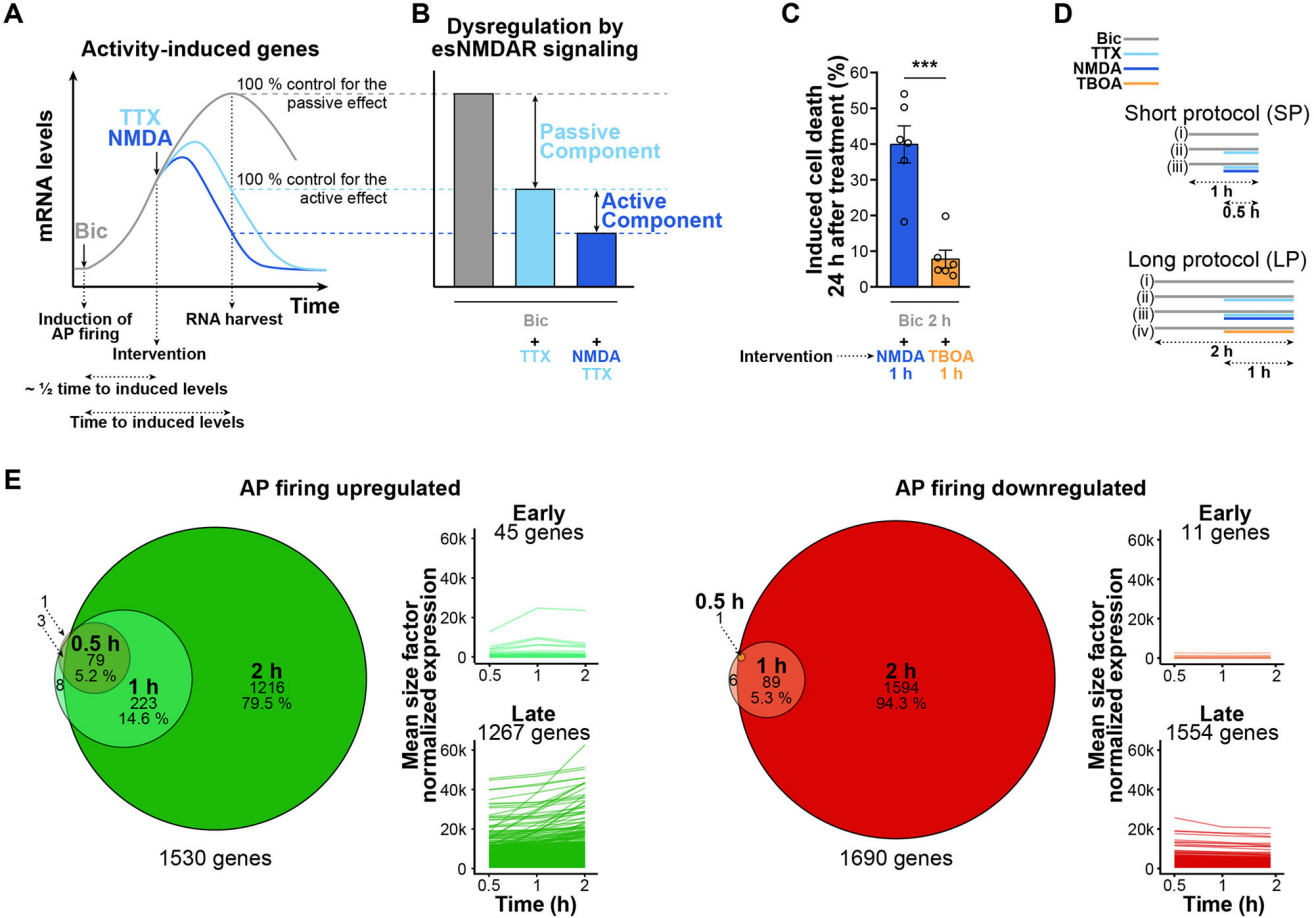
Principle for detecting dysregulated synaptic activity-induced gene expression by excitotoxic signaling in mouse hippocampal primary neurons. **A** Schematic of the theoretical expression levels over time of a typical activity-induced gene after initiation of strong excitatory synaptic activity in primary neuron cultures by bicuculline (Bic), which induces action potential (AP) firing, followed (or not) by interventions with tetrodotoxin (TTX) that blocks AP firing, and with high concentration (30 μM) of bath-applied N-methyl-D-aspartate (NMDA) that both silences APs (i.e., due to causing sustained membrane depolarization) and evokes excitotoxic signaling^30^. For capturing effects on synaptic activity-driven gene induction, we aimed to apply interventions before peak levels of expression, as indicated below the schematic. **B** Predicted relative activity-induced gene mRNA levels at the point of RNA harvest upon application of the excitotoxic interference protocol shown in (**A**). The difference in mRNA levels between Bic and Bic + TTX samples measures a passive component of transcriptional dysregulation, and the difference between Bic + TTX and Bic + TTX/NMDA samples measures an active component of excitotoxity-mediated transcriptional dysregulation. **C** Cell death evaluated by pyknotic nuclear Hoechst staining in mouse primary neuron cultures 24 h after induction of AP firing with Bic (50 µM), followed by an intervention with NMDA (30 µM) or DL-threo-β-benzyloxyaspartic acid (TBOA, 50 µM). *n* = 6 experiments on separate preparations. **D** Two AP firing interference protocols were devised for this study: a short protocol (SP), i.e., 1 h AP firing with a 0.5 h intervention; and a long protocol (LP), i.e., 2 h AP firing with a 1 h intervention. RNA samples for the indicated treatments (i), (ii), (iii), and (iv) (if applicable), and an untreated control RNA sample were collected (see “Materials and methods” for details). **E** Venn diagrams and line graphs show counts and counts with expression level trajectories, respectively, of synaptic activity-regulated genes. Differential gene expression analysis was performed using RNA-seq data obtained from primary neuron cultures that were either left untreated or were treated with Bic (50 µM) for 0.5, 1, or 2 h. Only genes displaying significantly up- or downregulated expression levels in Bic-treated samples compared to untreated samples are plotted. Responsive genes were categorized as being upregulated at “early” or “late” time points (significantly increased mRNA level maximum at 1 h or 2 h of AP firing, respectively), and as being downregulated at “early” or “late” time points (significantly decreased mRNA level minimum at 1 h or 2 h of AP firing, respectively). *n* = 5 experiments on separate preparations. **C** Mean ± SEM; ***p < 0.001; two-tailed *t*-test. **E** Differentially expressed genes cutoff FDR < 0.05; DESeq2 with Wald test and Benjamini–Hochberg correction.

The effects of esNMDAR activation during synaptic signaling generally have two components—(i) a passive component, i.e., due to inhibition of neuronal activity, and (ii) an active component, i.e., due to initiation of toxic signaling. In order to distinguish these two components at the level of transcriptional responses, we sought to establish a cell culture assay using mouse hippocampal primary neurons. We reasoned that induction of synchronized recurrent excitatory synaptic activity with the γ-aminobutyric acid (GABA) type A receptor antagonist bicuculline (Bic), which triggers activity-responsive gene expression^29^, followed by an intervention with either the sodium channel blocker tetrodotoxin (TTX), or by TTX in combination with bath application of NMDA (Fig. 1A), would provide robust selective read-outs for the passive and the active components of excitotoxic NMDAR stimulation effects on activity-dependent transcription, respectively (Fig. 1B). The intervention with TTX blocks action potential (AP) firing and thus models the termination of neuronal activity and sNMDAR activation^30^. The additional stimulation with bath-applied NMDA models the effect of toxic NMDAR signaling^30^ that is mediated by esNMDARs^5^. Accordingly, this study aims to firstly deduce the passive component of gene expression dysregulation associated with excitotoxic signaling by determining the difference between unaltered and TTX-interrupted AP firing-induced mRNA levels. Secondly, the active component will be identified by measuring the difference in transcript levels obtained when AP firing is interrupted by TTX alone from those obtained when AP firing is interrupted by TTX in the presence of bath-applied NMDA (Fig. 1B).

Bath application of NMDA to primary hippocampal neuron cultures is a pervasive and markedly severe analog of excitotoxic conditions in vivo. In order to additionally model milder, confined neurotoxic glutamate signaling that would more closely resemble excitotoxicity in neurodegenerative disorders, we used the excitatory amino acid transporter inhibitor DL-threo-β-benzyloxyaspartic acid (TBOA). During synaptic activity, TBOA forces a glutamate spillover^31^ that can activate esNMDARs in proximity to excitatory synapses. Notably, TBOA is less acutely toxic than NMDA in primary neuronal cultures, causing an increase in neuronal death of less than 10% when added after the initiation of AP firing with Bic compared to the 40% increase that is observed when NMDA is added (Fig. 1C).

The planned assay for detecting the dysregulation of synaptic activity-driven gene induction by excitotoxic signals requires that interventions affecting AP firing take place during periods of high ongoing transcription. This ongoing transcription is most likely to fall within time windows that, for the specific gene of interest, precede the point at which maximal gene expression levels are detected. Since synaptic activity-regulated genes respond to neuronal activity with different transcriptional kinetics, we decided to use two experimental designs that would target the two major waves of gene induction typically detected upon stimulating excitatory activity^4,32^. Firstly, for capturing the effects on rapidly induced genes, including most immediate early genes with peak mRNA expression within an hour after onset of AP firing (e.g., *Arc*), we devised a short protocol (SP) wherein the intervention is applied for 0.5 h after 0.5 h of AP firing (Fig. 1D). Secondly, for genes with delayed kinetics, such as *Bdnf*, we devised a long protocol (LP), wherein the intervention is applied for 1 h after 1 h of AP firing (Fig. 1D).

We used RNA sequencing (RNA-seq) with samples of neurons treated for 0.5, 1, and 2 h with Bic to verify that these induction durations permit the analysis of manipulation-dependent expression differences for the majority of synaptic activity-responsive genes at the chosen time points (Fig. 1E). In total, we detected 1530 upregulated (FDR < 0.05) genes upon AP firing. We filtered out those genes that were only very transiently upregulated (at the 0.5 h time point, but not at either 1 h or 2 h) and therefore not relevant for the planned assays. Genes that exhibited the highest transcript levels at 1 h were categorized as “early” upregulated genes and allocated to the SP analysis, while genes whose expression increased further between 1 and 2 h were categorized as “late” upregulated genes and allocated to the LP analysis (Fig. 1E). We estimate that almost 86% of the detected AP firing-induced genes (45 “early” and 1267 “late” upregulated, Supplementary Data 1) are covered by SP and LP assays for the detection of potential dysregulation by excitotoxicity. Of note, a similar number of genes was downregulated by AP firing as was upregulated (Fig. 1E, 11 “early” and 1554 “late” downregulated, Supplementary Data 1). Nevertheless, in this study, we focused on upregulated genes since activity-dependent gene induction is crucial for acquired neuroprotection and since overexpression of some of the induced genes has been shown to be sufficient for increasing neuronal resilience^2^.

### Excitotoxicity signature of the synaptic activity-regulated transcriptome

**Fig. 2:**
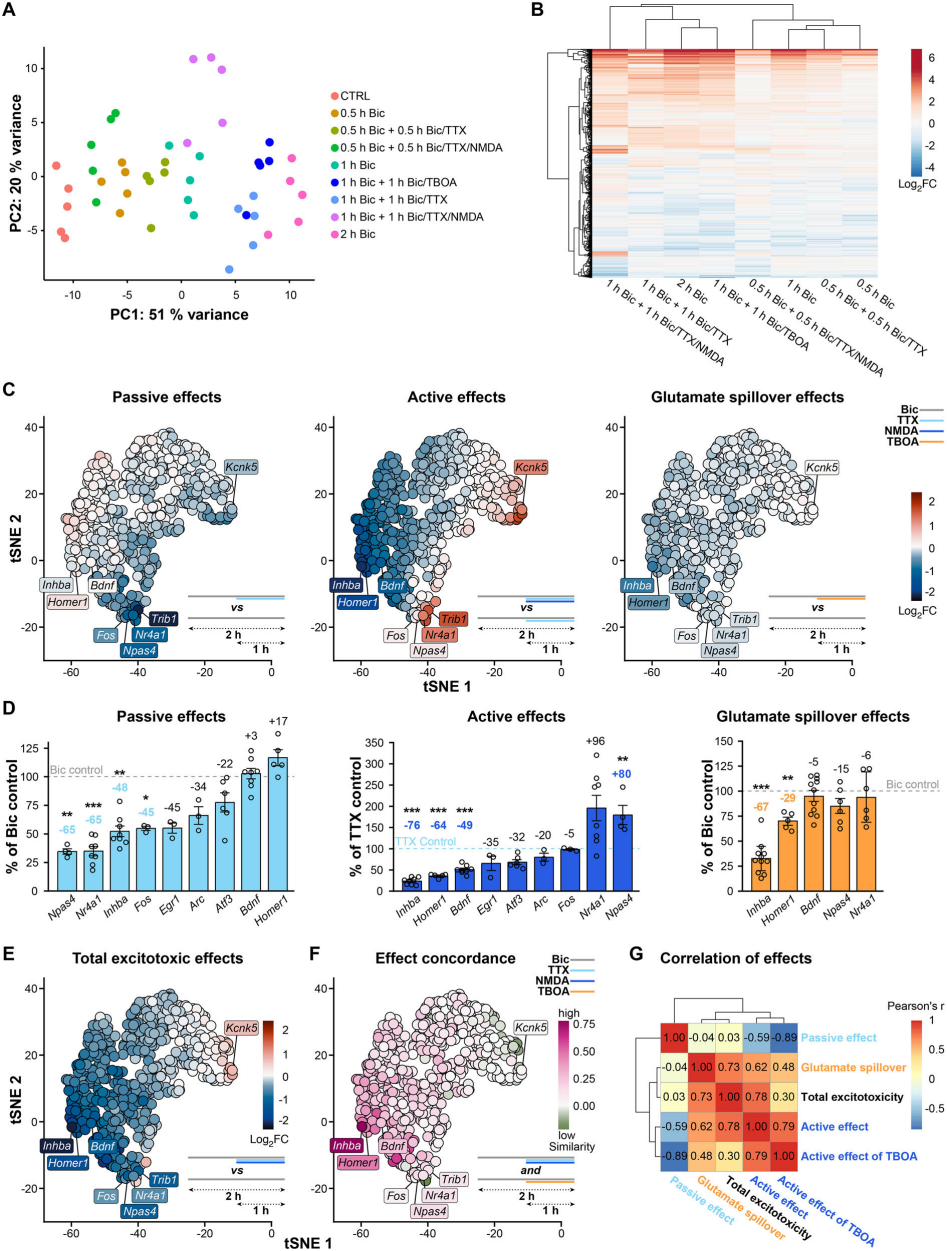
Dysregulation of transcriptomic responses to synaptic activity by excitotoxic esNMDAR signaling. Results of RNA-seq analysis of mouse hippocampal primary neurons treated according to protocols depicted in Fig. 1D. *n* = 5 experiments on separate preparations. **A** Principal component analysis of the expression profiles obtained for the indicated samples. **B** Heatmap of gene expression changes. Log_2_ fold change (FC) values relative to untreated controls, calculated with normalized read count means for each condition, were used to cluster genes and samples by Euclidean distance with complete linkage. 747 informative genes (rows) with log_2_FC standard deviation >0.5 across conditions were used. **C** t-SNE plots of “late” AP firing-upregulated genes embedding expression data of all the stimulation conditions applied. For discrete visualization of data points, only upregulated genes of the activity class “late.induced” are shown (569 genes, Supplementary Table 1B). Log_2_FC coloring is indicated in the legend together with the schematic representation of the applied treatments. **D** RT-qPCR-based quantifications and validations of the contribution of passive effects (by AP firing cessation with TTX) and active effects (via NMDAR stimulation) for esNMDAR signaling-evoked transcriptional dysregulation, and of the effect of glutamate spillover (by TBOA-mediated glutamate transporter block). Relative gene expression after SP or LP was measured, and the results for passive effects, active effects, and glutamate spillover are presented as the percentage of the mRNA levels in the indicated control condition. *n* = 3–10 experiments on separate preparations. *Bdnf*, *Bdnf* exon IV mRNA. **E**, **F** t-SNE plots as in (**C**). Log_2_FC coloring shows alterations in gene induction induced by Bic/NMDA/TTX as compared to Bic treatment (**E**). Gene-wise concordance of alterations in AP firing-induced gene expression evoked by Bic/TTX/NMDA as compared to Bic/TBOA (**F**). Log_2_FC values of the two conditions were used to calculate “similarity” that describes concordance in directionality and strength of changes (see “Materials and methods”). **G** Pearson correlation between gene induction alterations of “late” AP firing-upregulated genes. The following comparisons of induced gene expression levels obtained with the different conditions (LP, see Fig. 1C) were made: “Passive effect,” TTX vs control; “Glutamate spillover,” TBOA vs control; “Total excitotoxicity,” TTX/NMDA vs control; “Active effect,” TTX/NMDA vs TTX; “Active effect of TBOA,” TBOA vs TTX. t-SNE plots of the effects shown in (**C**) for AP firing “early” upregulated genes, as well as all downregulated genes, can be visualized with an online application (https://genome.leibniz-fli.de/shiny/shutoff/). **D** Mean ± SEM; significance levels were determined relative to the respective controls; *p < 0.05; **p < 0.01; ***p < 0.001; two-tailed one-sample *t*-tests with Bonferroni correction.

To get a comprehensive overview of how excitotoxicity impacts synaptic activity-regulated gene expression, we performed an RNA-seq analysis of mouse hippocampal primary neuron samples collected with the SP and LP assay protocols (see Fig. 1D). A principal component (PC) analysis of the transcriptomes showed that the duration of AP firing (Bic) correlates with PC1, which explains 51% of the expression variance in the experiment (Fig. 2A). AP firing blockade (Bic/TTX) shifted the expression patterns mostly along the PC1 axis, but when cells were challenged by the additional excitotoxic signal (Bic/TTX/NMDA) it shifted them also along PC2, demonstrating that the transcriptomes of those cells had changed distinctly. Glutamate spillover (Bic/TBOA) produced a small but clearly detectable shift of this sample cluster towards the Bic/TTX/NMDA group when compared to AP firing without interference, indicative of a similar but relatively weaker effect of the TBOA treatment (Fig. 2A). Data clustering by gene expression changes compared to untreated control confirmed that the excitotoxic intervention of AP firing caused divergent expression alterations to certain sets of genes. While shorter treatments (SP) evidently caused less pronounced expression changes than longer treatments (LP), Bic/TTX/NMDA samples differed from the others in both cases (Fig. 2B). Decreasing as well as increasing effects on inductions were detectable upon excitotoxic interventions, especially with the LP (Fig. 2B).

We zoomed in on the AP firing-upregulated genes in order to characterize transcriptional shut-off targets akin to *Bdnf*^5^. Notably, in addition to those genes that were upregulated by synaptic activity and whose expression was changed by excitotoxic stimulation, we also observed that a number of genes whose expression was unaltered by AP firing exhibited expression changes in response to the excitotoxic stimulation protocols (Supplementary Data 1). For the reasons described above, however, we focused specifically on the dysregulation of activity-driven gene induction. Thus, we first generated t-distributed stochastic neighbor embedding (t-SNE) plots of upregulated genes by merging expression data of all the conditions and color-coding the plots according to gene expression changes driven by the passive or the active effects of excitotoxicity, or by glutamate spillover (Fig. 2C, “late” upregulated genes; see https://genome.leibniz-fli.de/shiny/shutoff/ for an interactive online application that allows similar visualization of all data). Several patterns emerged from this analysis. Firstly, it was revealed that the expression of the most notable passive shut-off targets (e.g., *Npas4*, *Nr4a1*, and *Trib1*) was boosted by the NMDA-induced excitotoxic signal (Fig. 2C). Secondly, glutamate spillover, which is expected to engage both active and passive properties of excitotoxicity, generally decreased induction without the boosting effect of the NMDA treatment-mediated signaling seen for the passive shut-off target genes. And thirdly, the genes which were susceptible to active shut-off (e.g., *Bdnf*, *Inhba*, and *Homer1*) were mostly, albeit to a lesser extent, also negatively affected by glutamate spillover. Moreover, their activity-dependent induction appeared much less disturbed by simple AP firing termination (Fig. 2C, see also Supplementary Data 1), suggesting a dominant active shut-off mechanism acting on them.

We validated and quantified these findings by RT-qPCR for a set of example genes (Fig. 2D). Blocking of AP firing with TTX at assay midpoint indeed lowered *Npas4* and *Nr4a1* mRNA levels to 35% of their mRNA levels if measured from samples induced without intervention, confirming that they are targets of passive shut-off. *Inhba*, *Homer1* and *Bdnf* were very strongly affected by active shut-off mechanisms, as their mRNA levels were reduced 76%, 64% and 49%, respectively, by the NMDA/TTX intervention compared to the TTX-only intervention (Fig. 2D). As anticipated from the RNA-seq results, *Inhba* and *Homer1* inductions were also decreased by glutamate spillover, which significantly reduced their stimulated expression levels relative to uninterrupted AP firing, while *Bdnf*, *Npas4* and *Nr4a1* inductions were not affected (Fig. 2D). It is worth noting that the effect of TTX/NMDA intervention was not different from the effect observed with NMDA intervention alone (Supplementary Fig. 1).

Passive and active components of excitotoxicity differentially influence the expression of activity-induced genes, with gene induction alterations showing anticorrelated patterns (Fig. 2C, G; Pearson’s *r* (Passive effect, Active effect) = −0.59). However, with a few exceptions, the impact of active esNMDAR signaling largely predominates over the passive effect achieved by silencing synaptic activity, such that total excitotoxic effects are similar to the active effects (Fig. 2C, E, G; Pearson’s *r* (Total excitotoxicity, Active effect) = 0.78). Moreover, the consequences of AP firing intervention with TBOA, which stimulates glutamate spillover from synapses, resemble the induction-reducing effects of active esNMDAR signaling and correlate well with the total effect of acute excitotoxicity (Fig. 2F, G; Pearson’s *r* (Glutamate spillover, Active effect) = 0.62, and *r* (Glutamate spillover, Total excitotoxicity) = 0.73). This suggests that relatively weak esNMDAR activation that is subtoxic in short timescales (Fig. 1C), but potentially eventually lethal in chronic neurodegenerative diseases, involves active shut-off mechanisms of synaptic activity-responsive gene induction. The gene whose induction was most drastically reduced by excitotoxicity, being strongly affected both by bath-applied NMDA and spillover of synaptic glutamate, was *Inhba* (Fig. 2C, E, F), which encodes the prominent pro-survival protein inhibin β-A (monomer of activin A)^33,34^.

Together, these findings revealed that excitotoxic esNMDAR signaling, concurrent to glutamatergic synaptic activity, extensively and diversely perturbs activity-responsive gene expression, impacting approximately two-thirds of the activity-induced transcriptome. For details on specific genes, an interactive online application that allows the generation of bar plots of their expression across conditions is available (https://genome.leibniz-fli.de/shiny/shutoff/). The activity-induced genes affected most strongly by excitotoxicity are listed in Table 1.

**Table 1:** Synaptic activity-regulated genes most affected by excitotoxic signaling-associated transcriptional shut-off

Gene	Induction category	Shut-off %	Passive %		Active %		TBOA %		Neuroprotective (Ref)		Brain disorder association (Ref)
*Inhba*	late	−78.1	No		Yes	−76.4	Yes	−43.7	Yes	^64^	HD, ALS^23,63^
*Pim1*	late	−76.0	Yes	−50.5	Yes	−46.4	Yes	−34.2	Yes	^98^	
*Csrnp1*	late	−75.6	Yes	−35.4	Yes	−60.2	Yes	−36.2			
*Ptchd1*	late	−74.3	No		Yes	−72.3	Yes	−24.1			ID/ASD^99^
*Ankrd33b*	late	−73.0	No		Yes	−74.5	Yes	−28.9			
*Kcna1*	late	−72.7	No		Yes	−75.3	Yes	−23.4			EA1/epilepsy^100^
*Mir212*	late	−71.9	Yes	−37.1	Yes	−46.5	Yes	−35.5	Yes	^67^	AD^65^
*Rnf217*	late	−71.6	No		Yes	−74.8	Yes	−28.2			
*Frmd6*	late	−71.3	No		Yes	−68.4	Yes	−32.9	Yes	^101^	AD^101^
*Mir132*	late	−69.1	Yes	−33.3	Yes	−44.0	Yes	−37.1	Yes	^66^	AD^65^
*Sstr1*	late	−69.0	Yes	−22.8	Yes	−54.3	No		Yes	^102^	HD, AD^102,103^
*Sp7*	late	−68.8	No		Yes	−66.4	No				
*Homer1*	late	−65.9	No		Yes	−72.9	Yes	−28.6	Yes	^104^	AD, SCZ, TBI^105^
*Phf21b*	late	−65.0	No		Yes	−67.5	Yes	−25.9			MD^106^
*Shisa2*	late	−64.6	Yes	−28.1	Yes	−47.1	No				
*Stil*	late	−64.5	No		Yes	−69.7	Yes	−21.8	Yes	^107^	Microcephaly^108^
*Btg2*	early	−64.1	Yes	−29.7	Yes	−45.4			Yes	^109^	
*Zdbf2*	late	−63.8	No		Yes	−70.3	Yes	−15.7			
*Ccn1*	early	−63.3	Yes	−45.7	No				Yes	^110^	
*Pcdh8*	late	−63.3	Yes	−24.9	Yes	−49.8	Yes	−20.5			

“Induction category” specifies whether peak expression of the gene occurred early (1 h) or late (2 h) after induction of AP firing with bicuculline (50 μM) in mouse hippocampal primary cultures. “Shut-off %” represents the total induction reduction with NMDA/TTX intervention during AP firing. “Passive %” reflects induction reduction with TTX intervention compared to no intervention. “Active %” quantifies the induction reduction with NMDA/TTX intervention versus TTX intervention. “TBOA %” indicates induction reduction with TBOA intervention compared to no intervention. “Neuroprotective (Ref)” and “Brain disorder association (Ref)” show if the gene product is linked to neuroprotection or brain disorders, with example references provided.

The top 20 genes by shut-off % are shown.

*HD* Huntington’s disease, *ALS* amyotrophic lateral sclerosis, *ID* intellectual disability, *ASD* autism spectrum disorder, *EA1* episodic ataxia type 1, *AD* Alzheimer’s disease, *SCZ* schizophrenia, *TBI* traumatic brain injury, *MD* major depression.

### Selective susceptibility to esNMDAR signaling-mediated transcriptional shut-off

**Fig. 3:**
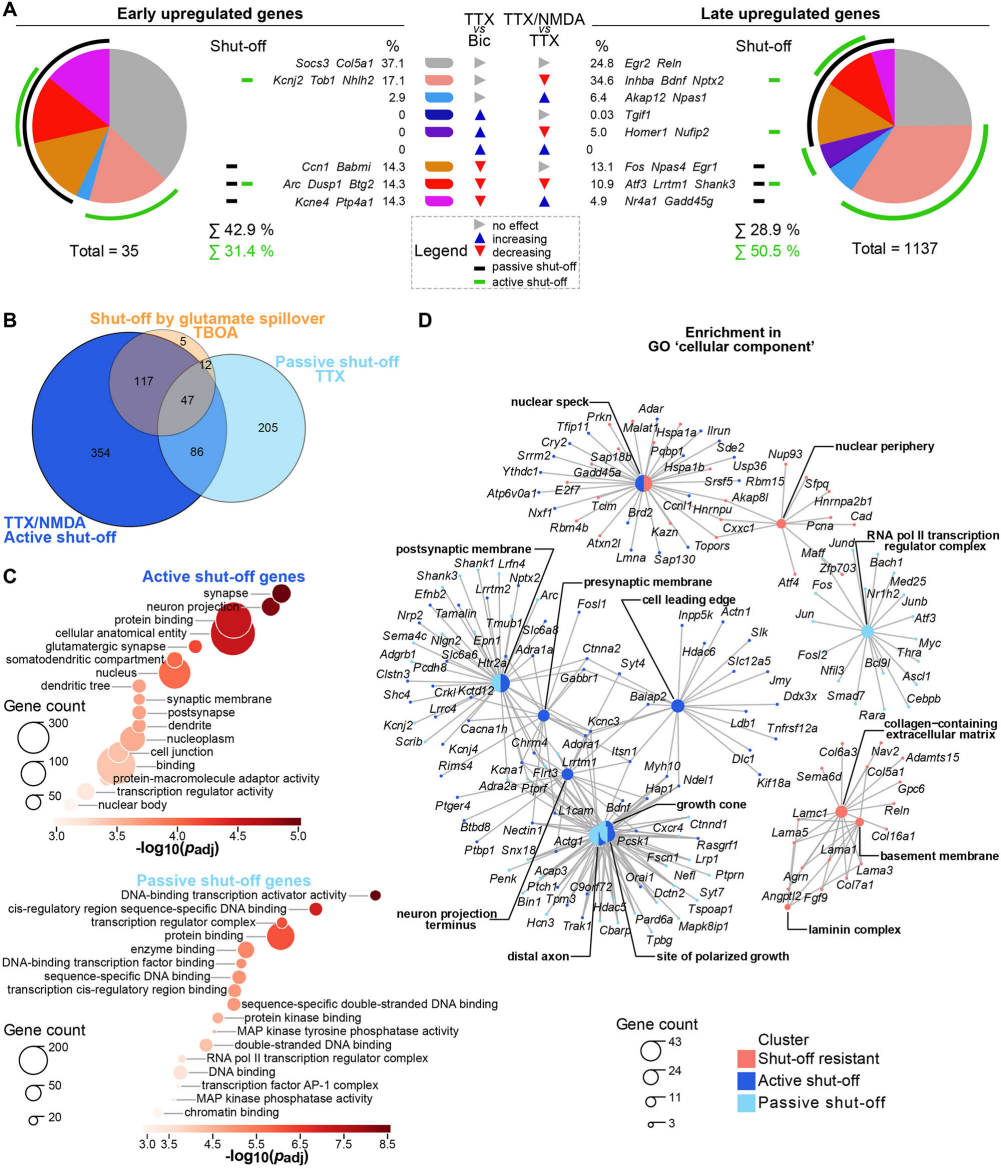
Characterization of the activity-induced gene set susceptible to esNMDAR-driven shut-off. **A** Pie charts showing proportions of “early” and “late” upregulated genes whose induction was either not affected, or was decreased or increased by TTX or TTX/NMDA intervention as indicated. “Shut-off” refers to a significantly decreased induction. Passive shut-off denotes that induction was significantly reduced by termination of AP firing (TTX). Active shut-off denotes that induction was significantly reduced by the additional excitotoxic signal instigated by NMDA (TTX/NMDA) compared to the induced mRNA levels obtained with the TTX-only intervention. Example genes are listed for every dysregulation class. **B** Venn diagram of synaptic activity-induced genes that display significantly reduced responses due to transcriptional shut-off initiated by glutamate spillover (TBOA), or due to passive (TTX) or active (TTX/NMDA) shut-off associated with esNMDAR activation. Gene counts belonging to each group are indicated. **C** GO term overrepresentation obtained with g:Profiler g:GOSt among all “early” and “late,” activity-induced genes that underwent active or passive shut-off upon excitotoxic signaling in mouse primary neurons. GO terms are from the sources “molecular function” and “cellular component”. For active shut-off genes, the most significantly enriched terms are shown; for passive shut-off genes, a selection from the top significance is shown to avoid highly redundant terms. Gene counts denote the intersection of genes allocated to the GO term and the query. Benjamini–Hochberg FDR correction (*p*_adj_) was used. **D** Net plot generated with clusterProfiler compareCluster of the top overrepresented GO terms from the source “cellular component” across shut-off resistant and active or passive shut-off susceptible AP firing-upregulated genes. Results with Benjamini–Hochberg FDR correction cutoff at 0.05 are shown.

We next sorted and counted the genes upregulated by synaptic activity according to their responses to TTX (i.e., passive effects) or TTX/NMDA intervention (comparison with TTX; i.e., active effects) (Fig. 3A). We took a conservative approach, categorizing only the genes for which statistical analysis results were available for all pair-wise condition comparisons (i.e., a gene was excluded if expression levels were not detectable in one or more conditions). We classified 35 “early” upregulated genes and found that the induction of the majority of them, measured at the 1 h time point, was susceptible to excitotoxicity-mediated shut-off (~43% to passive and ~31% to active). Five “early” upregulated genes, including the well-known activity-induced genes *Arc* and *Dusp1*, were affected by both passive and active shut-off (Fig. 3A, Supplementary Data 1). Among the 1137 “late” upregulated synaptic activity-responsive genes, the active NMDA-induced shut-off mechanism prevailed, as approximately half of these genes were induced to significantly lower levels upon the TTX/NMDA intervention compared to the TTX-only intervention. Moreover, most of the active shut-off targets (in total ~35% of all “late” upregulated genes, including *Bdnf*) were not significantly influenced by simple AP firing cessation. Still, a considerable fraction of the “late” upregulated genes (~29%) were passive shut-off targets as well. None of the synaptic activity-induced genes were boosted by both AP firing cessation and esNMDAR signaling, although some genes were more strongly induced with the TTX-only or the TTX/NMDA intervention (Fig. 3A, Supplementary Data 1).

To explore the potential functional significance of distorted activity-responsive gene induction caused by excitotoxic conditions, we used gene ontology (GO) analyses. As was evident from the t-SNE plots and correlation analysis (Fig. 2C, F, G), glutamate spillover during excitatory synaptic activity causes a milder version of active esNMDAR signaling-mediated gene dysregulation that was modeled here by the TTX/NMDA intervention. Specifically, 91% (164 out of 181) of the TBOA intervention-affected genes overlapped with those impacted by TTX/NMDA (Fig. 3B). We therefore concentrated on the active and passive shut-off targets, defined here via TTX/NMDA and TTX interventions, that contained more genes (604 and 350, respectively) and were partially overlapping (133 genes in common, Fig. 3B).

GO term overrepresentation analysis showed that activity-induced genes that are susceptible to shut-off by excitotoxic esNMDAR signaling comprise a substantial portion of genes encoding synaptic proteins, particularly those that function in glutamatergic synapses (Fig. 3C). Active shut-off also affected the induction of a diverse set of activity-regulated nuclear protein-encoding genes. Passive shut-off primarily inhibited activity-dependent upregulation of sequence-specific DNA-binding transcription factors or transcriptional regulators (Fig. 3C). To gain deeper insight into the cellular dynamics potentially influenced by the observed changes in gene expression profiles, we compared GO term enrichments among gene clusters formed by differential responses to AP firing interventions. In this analysis, the most revealing with respect to term distribution were enrichments within the GO source “cellular component” (Fig. 3D). The results provided further evidence that transcriptional regulation may be perturbed specifically by the passive termination in AP firing. In particular, the functional and/or structural dysregulation of synaptic membranes, likely involving their activity-dependent plasticity, appears to be a general outcome of excitotoxicity, since enrichment of terms such as “postsynaptic membrane,” “growth cone,” and “site of polarized growth” were shared by active and passive shut-off gene clusters (Fig. 3D). Active esNMDAR signaling during synaptic activity may, on the other hand, have more specific effects on presynaptic terminals (Fig. 3D, enriched terms “presynaptic membrane,” “cell leading edge,” and “neuron projection terminus”). Into this analysis, we included the part of the activity-induced gene program that, according to our RNA-seq results, was resistant to excitotoxic shut-off mechanisms. Enrichments among this group of genes revealed that the activity-driven gene expression response that targets the extracellular milieu may remain comparatively intact in excitotoxic conditions (enriched terms, e.g., “collagen-containing extracellular matrix” and “basement membrane”). Also, activity-regulated changes to splicing factor abundancies may partially be protected (Fig. 3D, enriched term “nuclear speck”).

Together, these results show that excitotoxicity may cause ineffective upregulation of transcription regulatory protein expression by silencing AP generation. Moreover, they indicate that excitotoxic conditions additionally diminish the induction of genes encoding synaptic proteins primarily via active esNMDAR signaling. The active and passive shut-off effects combined could thus substantially downgrade the adaptive, synaptic plasticity-promoting function of the activity-driven genomic response.

### ERK/MAPK-ELK1/SRF pathway is suppressed by excitotoxic esNMDAR signaling

A key event in the esNMDAR signaling-driven reduction of activity-dependent *Bdnf* induction is CREB shut-off^5^. Indeed, calcium signaling through calcium/calmodulin (CaM)-dependent protein kinase IV (CaMKIV) to CREB and CREB binding protein (CBP) is the central mechanism that controls activity-induced gene expression^3,35,36^, including the induction of such genes as *Bdnf*^37–39^. The second major branch of synapse-to-nucleus signaling is the Ras/extracellular-signal-regulated kinase (ERK)/mitogen-activated protein kinase (MAPK) cascade^40^ that phosphorylates ETS-like protein 1 (ELK1), a ternary complex factor required for serum response factor (SRF)-dependent transcription^35,41^, which controls, in part, the induction of *Arc* and *Egr1*^42^, for example. Because negative regulation of ERK function by esNMDAR activity has been demonstrated^43^, it is likely that esNMDAR signaling during synaptic activity also affects the ERK-ELK1 pathway. Thus, we next used the AP firing intervention protocol to analyze by immunoblotting the phosphorylation statuses of ERK1/2 and ELK1, and, as a control, CREB. Since synaptic activity-stimulated intracellular signaling cascades are rapidly induced and short-lived (see e.g.,^43–45^), we reduced the experiment duration to a total of 20 min, using a 10-min intervention protocol. Also, we included the condition with NMDA intervention alone to investigate whether the effects of esNMDAR stimulation with or without TTX addition are comparable.

**Fig. 4:**
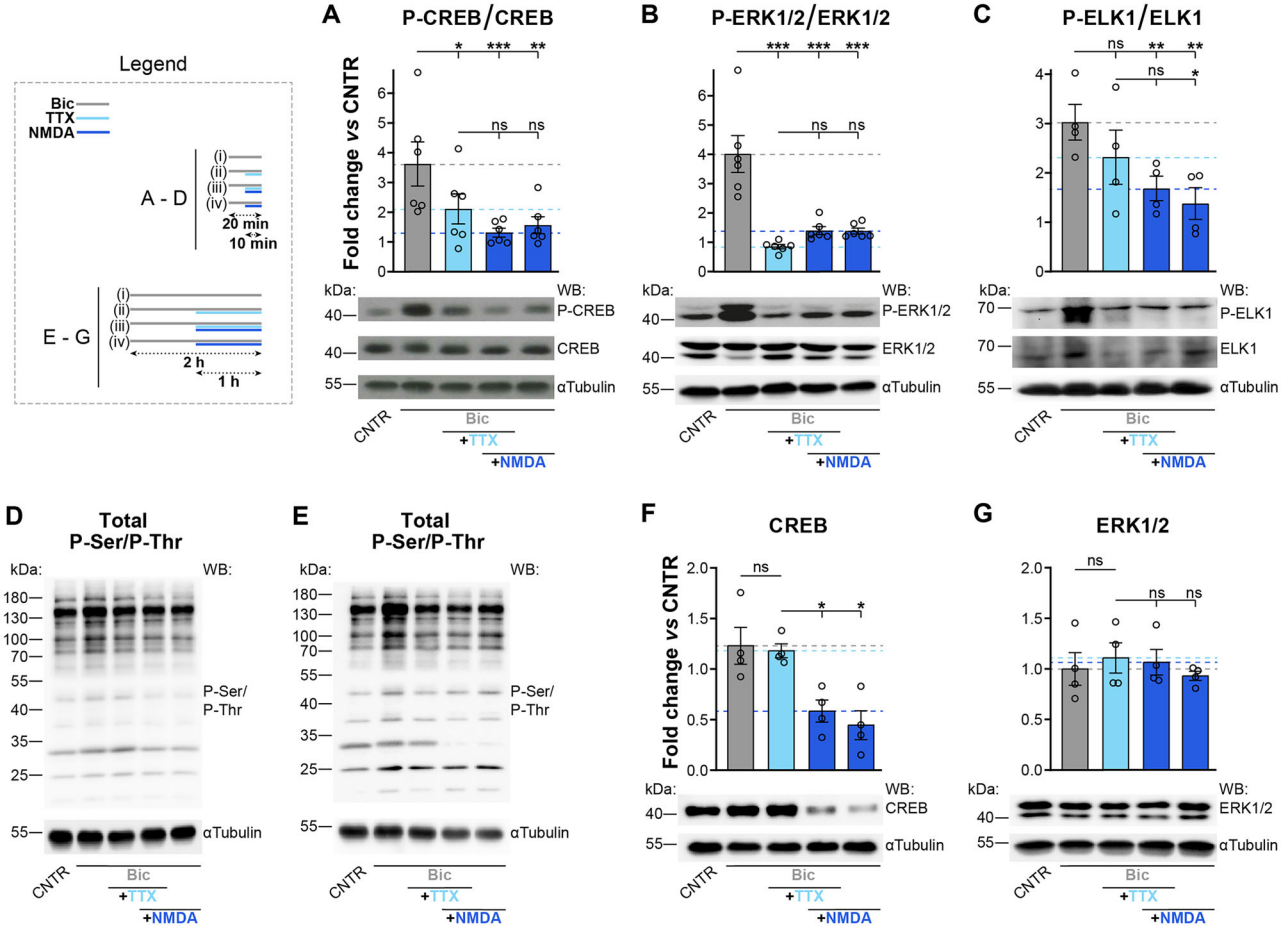
Passive and active excitotoxicity-associated suppression of CREB, ERK1/2, and ELK1. Mouse hippocampal primary neuron cultures were left untreated or were treated as indicated in the panels and in the legend with (i), (ii), (iii), and (iv) (see “Materials and methods” for details). Western blotting was performed. **A**–**C** Quantifications and representative immunoblots of the phosphorylation statuses of functionally relevant sites for the designated proteins. Phosphorylation levels normalized to the total levels of the respective protein are shown. The 20 min treatment protocol was applied. *n* = 4–6 experiments on separate preparations. **D**, **E** Representative immunoblots of total serine and threonine phosphorylation (P-Ser and P-Thr, respectively). The 20 min protocol (**D**) and LP (**E**) were used. **F**, **G** Quantifications and representative immunoblots of CREB and ERK1/2 expression levels. Quantifications are normalized to αtubulin. The LP was applied. *n* = 4 experiments on separate preparations. **A**–**C**, **F**, **G**, Mean ± SEM; ns not significant, *p < 0.05; **p < 0.01; ***p < 0.001; RM one-way ANOVAs with Tukey’s tests. kDa kilodalton, WB Western blotting with antibodies against the indicated targets.

We detected a reduction in CREB phosphorylation (Ser 133) with TTX and a more pronounced reduction following interventions with either NMDA or TTX/NMDA (Fig. 4A), indicating that passive and active shut-off components were both at play. ERK1/2 phosphorylation (Thr 202/Tyr 204) was greatly reduced by all of the applied interventions, with TTX addition during AP firing producing the largest effect (Fig. 4B). Phosphorylation of ELK1 (Ser 383) was not affected by terminating AP firing with TTX, but was strongly reduced when TTX/NMDA or NMDA were applied (Fig. 4C), suggesting that ELK1 phosphorylation is relatively stable and needs strong stimulated activity of a phosphatase, probably calcineurin^46,47^, for inactivation. In sum, these results show that esNMDAR stimulation during synaptic activity can silence both major branches of intracellular synapse-to-nucleus signaling pathways.

To exclude the possibility that excitotoxicity-induced protein dephosphorylation occurs globally rather than target-specifically, we analyzed the total Ser/Thr phosphorylation status in the cell lysates obtained with the AP firing intervention protocols. We found that 20 min, but also 2 h of AP bursting caused small increases in overall phosphorylation, and interventions with TTX, TTX/NMDA, or NMDA did not result in any obvious decrease in phosphorylation (Fig. 4D, E). Moreover, to exemplify that the reported excitotoxicity-associated degradation of CREB^48^ is specific to CREB, we analyzed the expression of total CREB and ERK1/2. We found that while ERK1/2 levels remained stable in all conditions, CREB levels declined after 1 h, but not after 10 min, of active esNMDAR signaling (TTX/NMDA or NMDA) (Fig. 4F, G, 1 h treatment; see Fig. 4A, B for the 10 min treatment).

These results indicate that in addition to CREB shut-off, esNMDAR signaling inhibits ERK1/2 and ELK1 activities by reducing their phosphorylation.

### Memantine partially inhibits excitotoxicity-associated shut-off of *Inhba* and *Bdnf*

**Fig. 5:**
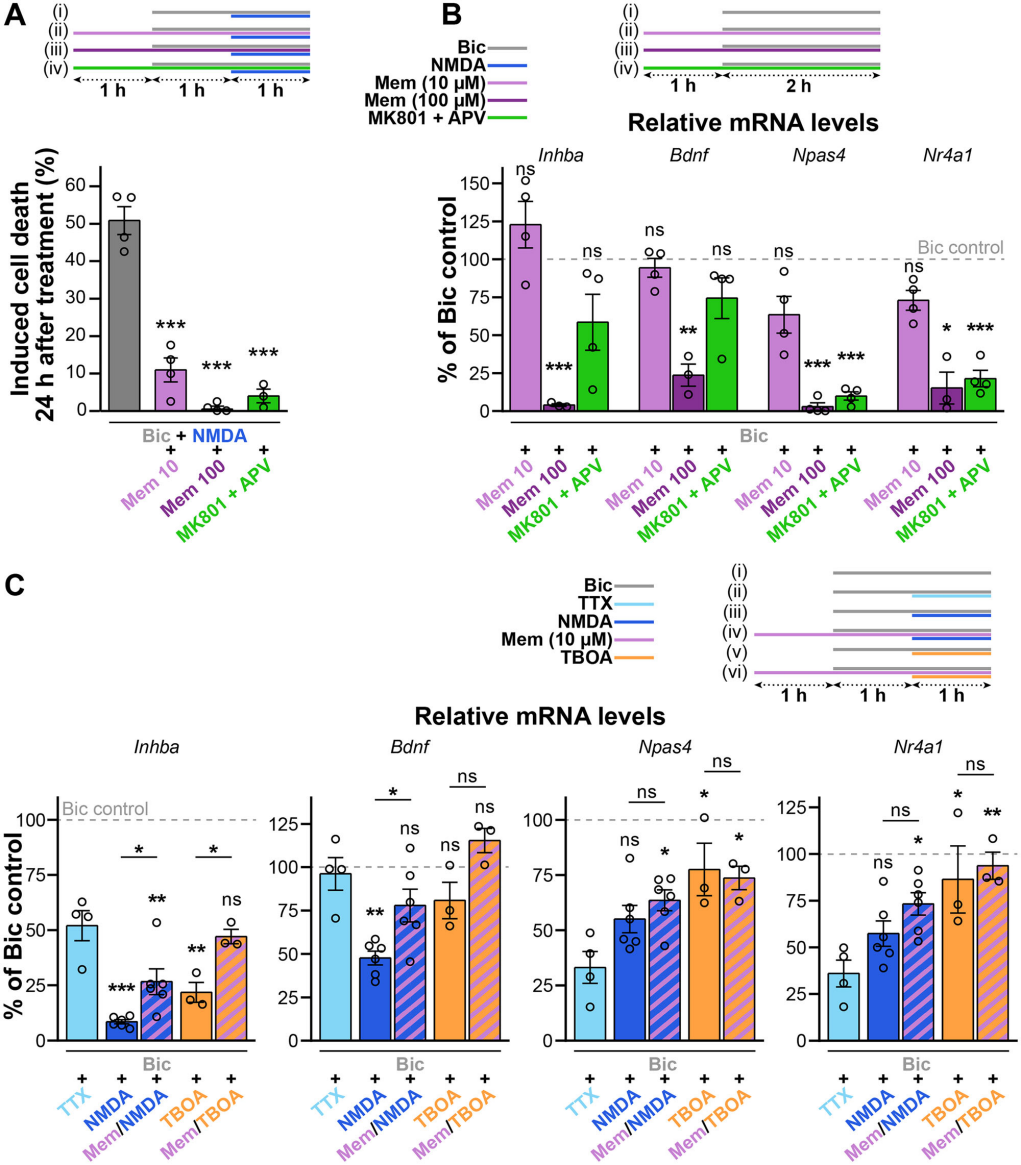
Rescue of excitotoxicity-associated activity-driven dyregulation of gene induction by the NMDAR antagonist memantine. AP firing was induced in mouse primary neuron cultures with the addition of bicuculline (Bic, 50 µM) for the conditions specified within the schematics. The effects of the low-affinity uncompetitive NMDAR antagonist memantine (Mem; 10 or 100 µM) or the NMDAR open-channel blocker MK801 (10 µM) and the competitive NMDAR inhibitor 2-amino-5-phosphonopentanoic acid (APV, 50 µM) were assessed. **A** Cell death evaluated by pyknotic nuclear Hoechst staining in mouse primary neuron cultures 24 h after an excitotoxic insult (NMDA, 30 µM). *n* = 3–4 experiments on separate preparations.Quantifications of the effects of NMDAR inhibition on AP firing-dependent gene induction (**B**) or on esNMDAR signaling-evoked dysregulation of AP firing-dependent gene induction (**C**). NMDA (30 µM) and TBOA (50 µM) model strong and mild excitotoxic conditions, respectively; TTX (1 µM) intervention is shown for comparison as the passive effect of excitotoxic signaling (**C**). RT-qPCR results are presented as the percentage of the mRNA levels measured in samples treated with Bic only (Bic, 50 µM); *n* = 3–4 (**B**) or *n* = 3–6 (**C**) experiments on separate preparations. *Bdnf*, *Bdnf* exon IV mRNA. **A**–**C**, Mean ± SEM; significances are relative to controls or as indicated with lines; ns not significant, *p < 0.05; **p < 0.01; ***p < 0.001; one-way ANOVAs with Dunnett’s (**A**) or Šidák’s (**C**) tests; two-tailed one-sample *t*-tests with Bonferroni correction (**B**).

Given that excitotoxic pathology is observed in various neurodegenerative disorders^7^, we next asked if it is possible to ameliorate the excitotoxicity-driven shut-off of synaptic activity-driven gene expression by antagonizing esNMDAR signaling in primary neuron cultures. A suitable treatment would ideally block esNMDARs and thereby save neurons from dying while leaving sNMDAR signaling and activity-driven gene induction unaffected. We selected memantine, an NMDAR open-channel blocker that preferentially inhibits esNMDARs independent of NMDAR subunit composition^26,49^. However, since memantine can also interfere with sNMDAR function at higher concentrations, we compared its capacity to inhibit excitotoxic cell death and its compatibility with activity-regulated transcription at a low (10 µM) and a high (100 µM) concentration. As a control for complete NMDAR inhibition, we included APV, a competitive NMDAR inhibitor, in combination with MK801, an NMDAR open-channel blocker. Our results showed that when primary hippocampal cells were challenged with NMDA after 1 h of AP firing, all the tested NMDAR inhibition conditions efficiently prevented neuronal death (Fig. 5A). However, while the presence of 10 µM memantine allowed for nearly unaltered induction of *Inhba*, *Bdnf*, *Npas4* and *Nr4a1*, the high dose strongly inhibited activity-dependent expression of these genes (Fig. 5B). APV/MK801 reduced *Npas4* and *Nr4a1* inductions, but did not significantly lower *Inhba* and *Bdnf* upregulation (Fig. 5B), possibly because of relatively larger contribution of other calcium sources, such as VGCCs^50,51^, to the transcriptional activation of these genes.

Next, we investigated the potential of memantine to interfere with the esNMDAR signaling-induced shut-off of activity-dependent gene induction in excitotoxic conditions. We applied the LP with or without 10 µM memantine and used both NMDA and TBOA for modeling strong and mild excitotoxic conditions, respectively. We found that *Inhba* induction was significantly decreased by the active component of the esNMDAR signaling-associated shut-off (NMDA, <10% of Bic-only control) and by glutamate spillover (TBOA, ~20% of Bic-only control), and that *Inhba* induction in these conditions was partially or fully rescued by memantine, respectively (Fig. 5C). Similarly, NMDA intervention of AP firing resulted in the expected shut-off of *Bdnf* induction, which was prevented in the presence of memantine (Fig. 5C). TBOA did not significantly affect *Bdnf* expression (Fig. 5C). *Npas4* and *Nr4a1*, targets of the passive shut-off (see Fig. 2C, D), responded to AP firing and subsequent NMDA or TBOA interventions with slightly increased inductions, which were not affected by memantine (Fig. 5C). These results indicate that memantine at a low concentration can save neurons from death in excitotoxic conditions without significantly compromising activity-dependent transcription. In addition, memantine can at least partially protect neurons from the excitotoxicity-related active transcriptional shut-off process that is stimulated by esNMDAR signaling.

### Inhibition of esNMDAR signaling restores normal expression levels of downregulated activity-responsive genes in a mouse model of Huntington’s disease

**Fig. 6:**
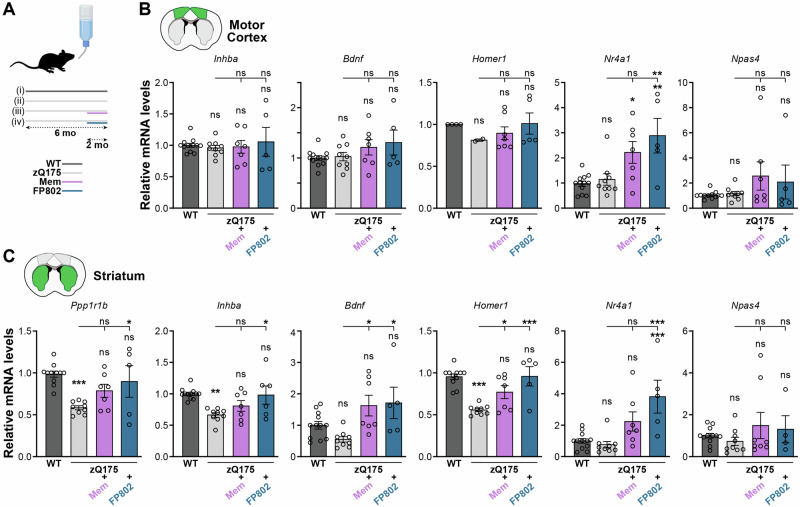
Rescue of transcriptional dysregulation in a mouse model of Huntington’s disease with esNMDAR signaling inhibitors. **A** Schematic of the protocol. Huntington’s disease model mice whose *Htt* gene exon 1 is replaced by the human *HTT* exon 1 sequence with an expanded CAG repeat tract (zQ175) were left untreated or were dosed via the drinking water for 2 months (mo) with either memantine (Mem 10 mg/kg/day) or the TwinF interface inhibitor FP802 (40 mg/kg/day). Untreated wild-type (WT) mice served as a reference control. Image was created in https://BioRender.com. RT-qPCR results of relative mRNA levels of the indicated genes in the motor cortex (**B**) and striatum (caudate putamen) (**C**) after the treatment regimen specified in (**A**). WT, *n* = 12; zQ175, *n* = 9; zQ175/Mem, *n* = 7; zQ175/FP802, *n* = 5 mice. *Bdnf*, *Bdnf* exon IV mRNA. **B**, **C**, Mean ± SEM; significances are relative to WT control or as indicated with lines; ns not significant, *p < 0.05; **p < 0.01; ***p < 0.001; one-way ANOVAs with Tukey’s tests.

Toxic esNMDAR signaling and CREB shut-off are part of the pathogenic mechanism in HD^17,52^, raising the possibility that dysregulation of activity-responsive gene expression may contribute to the disorder. To assess the potential of inhibiting toxic signaling originating from esNMDARs to normalize such dysregulation, we used the zQ175 mouse model of HD, which harbors an expanded CAG repeat knock-in in its *Htt* gene^53^. Mice were subjected to a 2-month oral administration regime of either memantine or the TwinF interface inhibitor, FP802, a recently developed small molecule neuroprotectant that detoxifies esNMDARs^27^ (Fig. 6A). FP802 disrupts the interaction of NMDARs with TRPM4, which is responsible for the toxic properties of esNMDARs^27^. Recent studies have shown the therapeutic potential of FP802 in the SOD1^G93A^ mouse model of ALS, where it stopped the degeneration of spinal motor neurons and retinal ganglion cells^22,23^. Guided by our transcriptomic results with cultured cells (Figs. 2 and 5), we investigated the expression of prominent active shut-off and passive shut-off targets, performing RT-qPCR analysis of *Inhba*, *Bdnf*, and *Homer1*, as well as *Nr4a1* and *Npas4* expression in the striatum and the motor cortex, the two brain regions primarily affected in HD^54^. To validate the model, we first analyzed striatal expression of *Ppp1r1b* (encoding dopamine- and cAMP-regulated phosphoprotein, 32 kDa; DARPP-32), a medium spiny neuron marker, the downregulation of which correlates with disease progression^55,56^. We detected a reduction of *Ppp1r1b* mRNA levels in the striatum of zQ175 mice, indicative of HD-associated striatal pathology (Fig. 6C). Treatment of zQ175 mice with FP802 rescued the expression levels of *Ppp1r1b* (Fig. 6C), suggesting that FP802 can mitigate HD disease progression. We also detected a trend—albeit not statistically significant—towards increased levels of *Ppp1r1b* in zQ175 mice treated with memantine compared to controls (one-way analysis of variance (ANOVA) with Tukey’s test, *p* = 0.2; Fig. 6C).

We next analyzed the expression of the selected set of activity-regulated genes in wild-type and zQ175 control mice, and in zQ175 mice treated with memantine or FP802. Compared to wild-type mice, the mRNA levels of the active shut-off targets *Inhba*, *Homer1*, and *Bdnf* were unaltered in the cortex of zQ175 mice, but downregulated in the striatum (Fig. 6B, C), although the reduction of *Bdnf* expression did not reach statistical significance (one-way ANOVA with Tukey’s test, *p* = 0.4). There were no differences in transcript levels of the passive shut-off targets *Nr4a1* and *Npas4* between zQ175 and wild-type mice in either brain region. Treatment of zQ175 mice with FP802 rescued striatal expression of *Inhba*, *Bdnf*, and *Homer1*; memantine treatment rescued striatal expression of *Bdnf* and *Homer1* (Fig. 6C). Compared to wild-type and untreated zQ175 mice, we observed higher mRNA levels of *Nr4a1* in both the striatum and the cortex of zQ175 mice treated with either memantine or FP802 (Fig. 6B, C). *Npas4* mRNA levels varied greatly, and no significant differences across treatment conditions were revealed. These results indicate that memantine and, to an even greater extent, FP802 are effective in rescuing the esNMDAR signaling-mediated downregulation of synaptic activity-responsive genes in vivo in a mouse model of neurodegenerative disease.

## Discussion

Our study uncovered a distinct pattern of dysregulation in neuronal activity-dependent gene expression under conditions of excitotoxic esNMDAR signaling. This result is indicative of disrupted adaptive transcriptional programs important for synaptic functions and neuronal resilience, which are negatively affected in neurodegenerative diseases. While confirming the central role of CREB shut-off as a key mediator of this transcriptional impairment, we also identify the inactivation of the ERK/MAPK-ELK1/SRF signaling axis, which is critical for SRF-dependent gene induction, as an additional consequence of esNMDAR signaling. Among the affected activity-regulated genes, *Inhba* exhibits the most pronounced suppression in response to excitotoxicity. Its repression, along with the impairment of *Bdnf* and *Homer1* induction, is prevented by treatment with the NMDAR blocker memantine and the TwinF interface inhibitor FP802. These findings not only highlight the central role of esNMDAR-mediated signaling in disrupting neuroprotective transcriptional responses but also underscore a promising therapeutic strategy for counteracting transcriptional deficits in neurodegenerative conditions.

### Reduced levels of *Inhba* may drive a feedforward loop amplifying toxic esNMDAR signaling

Our results demonstrate that a large fraction of the synaptic activity-regulated transcriptome is perturbed by neurodegenerative disease-associated excitotoxic signaling. The scale of the effect is in line with the shut-off of both CREB-dependent and ELK1/SRF-dependent transcription, and possibly additionally comprises a repressive contribution from nuclear accumulation of class IIa histone deacetylases (HDACs)^57–59^. Genes involved in synaptic function and gene regulation appear broadly affected, and a substantial number of neuroprotective genes are among the top targets hit by acute esNMDAR-mediated shut-off in primary neuron cultures, including *Inhba*, *Atf3*, *Bdnf*, and *Btg2* (^2,60,61^; see also Table 1). The gene most severely and consistently suppressed by excitotoxicity was *Inhba*, which encodes the pro-survival protein inhibin β-A (two subunits of inhibin β-A form activin A)^33,34,62^. The loss of activity-dependent expression of *Inhba* may act as a driver of a deleterious feedforward loop in which toxic esNMDAR signaling becomes sustained and ultimately overrides neuroprotective sNMDAR activity, thereby promoting neurodegeneration. In particular, in conditions of *Inhba* downregulation, esNMDAR activity is heightened^63^, potentially due to a role of inhibin β-A/activin A in limiting cell surface expression of NMDAR^64^. Thus, reduced levels of *Inhba* may promote increased surface expression of esNMDARs, amplifying harmful signaling and reinforcing a vicious cycle that culminates in neuronal damage. A concomitant shut-off of *Bdnf* induction further compromises the intrinsic ability of neurons to withstand excitotoxic insults. Reduced Bdnf levels diminish the efficacy of synaptic activity-driven, transcription-dependent acquired neuroprotection^64^, a mechanism supported not only by *Inhba*, but also by *Bdnf* itself^60,61,64^, as well as other survival-promoting genes, including *Atf3* and *Btg2*^2^. The activity-driven neuroprotective gene program also includes microRNAs *miR-132* and *miR-212*^65–67^, whose expression is induced by survival-promoting sNMDAR signaling^68^, but repressed by esNMDAR activity (see Table 1). Consequently, the excitotoxic component of a neurological disorder may be exacerbated by a sustained failure to suppress toxic esNMDAR activity due to insufficient levels of inhibin β-A, coupled with a simultaneous reduction in pro-survival sNMDAR signaling due to Bdnf deficiency. This dual impairment leads to a progressive decline in neuronal resilience. A pertinent example is HD, where the supply of Bdnf from the cortex to striatal neurons is reduced^69–74^. This deficit, when combined with the striatal downregulation of *Inhba* observed in the present study (see Fig. 6C), could amplify excitotoxic vulnerability^63^. Notably, restoration of *Inhba* levels in HD mice using viral vector-mediated gene therapy has been shown to rescue key aspects of HD pathology^63^.

### Consequences of transcriptional shut-off induced by esNMDARs for neuronal health and network functions

We show that excitotoxic signaling disrupts neuronal activity-responsive gene regulation through both passive and active mechanisms. By distinguishing between termination of synaptic activity and active engagement of esNMDAR signaling, we uncovered distinct transcriptomic consequences that may underlie different patterns of dysfunction during the progression of a neurodegenerative disorder. Our data indicate that reduced synaptic activity preferentially impairs the induction of key transcription factors, such as *Fos*, *Npas4*, and *Egr1*, which are known to be involved in cell type-specific regulation of circuit plasticity and maintenance of excitatory-inhibitory balance^75–78^. Failure in these mechanisms may contribute to inhibitory dysfunction and network abnormalities, including hyperexcitability that has been detected already in presymptomatic phases of AD, HD, and ALS^79–82^. In early stages of neurodegenerative diseases, the detrimental effects of esNMDAR signaling may still largely be counterbalanced by synaptic activity-driven neuroprotective mechanisms. However, these mechanisms will deteriorate when esNMDAR signaling intensifies and overrides synaptic signaling, causing deficits in activity-responsive gene products involved in synaptic functions (e.g., *Homer1*, *Lrrtm1*, *Baiap2*, *Chrm4*, *Nptx2, Bdnf*) and neuronal survival (e.g., *Bdnf*, *Inhba*, *Mir132*, *Mir212*, *Dusp1*), as reflected in the esNMDAR-driven active shut-off targets identified in this study. While additional shared and disease-specific processes undoubtedly contribute to the etiologies of neurodegenerative diseases (e.g^83–86^), our findings suggest a common mechanism: dysregulation of vital synaptic activity-driven gene expression by active, esNMDAR-mediated transcriptional shut-off. This mechanism may significantly contribute to altered network connectivity and death of neurons in prodromal and advanced stages of neurodegenerative diseases^87–91^.

### Therapeutic potential of targeting esNMDAR signaling in a mouse model of Huntington’s disease—rescue of *Inhba* and disease marker expression

Oral administration of memantine or FP802 to target esNMDAR signaling in vivo prevented the dysregulation of the activity-responsive genes *Inhba*, *Bdnf*, and *Homer1* in the striatum of a mouse model of HD. FP802 also restored expression of the disease marker, *Ppp1r1b* (see Fig. 6C), indicating its potential to mitigate HD disease progression. Memantine has previously been shown to improve the pathology in HD mice^17^; it can also provide therapeutic benefits in patients suffering from AD^92^. FP802, a derivative of the recently discovered TwinF interface inhibitor, compound 8^27^ represents a novel class of neuroprotectants. It was shown to prevent death of both motor neurons and retinal ganglion cells in the SOD1^G93A^ mouse model of ALS, leading to improved motor performance, an extension of lifespan, and improved central retinal function^22,23^. The results presented here align with the neuroprotective effects of this new drug class in excitotoxic conditions and support the therapeutic potential of FP802 in HD. Interestingly, we observed decreased *Inhba* and *Bdnf* transcript levels not in the cortex, but in the striata of zQ175 HD mice (see Fig. 6). This finding contrasts with the prevailing hypothesis that reduced cortical supply of the neurotrophin BDNF underlies striatal vulnerability in HD^63,69,70,73^. However, the downregulation in zQ175 mice of *Ppp1r1b* mRNA, an established early marker of striatal degeneration^55,56^, and its rescue by FP802, suggest that excessive esNMDAR signaling has a direct and deleterious impact on striatal health in the HD mice. Reduced expression of *Inhba* and *Homer1*, as well as other esNMDAR signaling-associated transcriptional shut-off targets, such as *Csrnp1*, *Ankrd33b*, and *Frmd6* (see also Table 1 and Supplementary Data 1), may thus serve as general markers of excitotoxicity-related neuropathology. Supporting this notion, *Ppp1r1b* was also significantly downregulated by esNMDAR activation in primary hippocampal neurons (−30%, *p*_adj_ < 0.01, Bic/TTX/NMDA vs Bic at the 2 h time point; Supplementary Data 1).

### Conclusion and implications

Our findings reveal a previously underappreciated mechanism by which glutamate excitotoxicity disrupts neuronal resilience: active transcriptional repression induced by toxic esNMDAR signaling. By identifying a transcriptome signature of downregulated synaptic activity-responsive genes that include regulators of synaptic function and neuronal survival, we defined a molecular footprint of esNMDAR signaling with implications across neurodegenerative diseases. Although first identified in cultured hippocampal neurons and validated in vivo in a mouse model of HD, esNMDAR-driven transcriptional dysregulation likely extends to other neurodegenerative diseases, including AD and ALS, where esNMDAR signaling has also been implicated. The recovery of protective gene expression through pharmacological inhibition of esNMDAR activity highlights the possible clinical relevance of these findings. Moreover, esNMDAR-regulated genes may serve as biomarkers for excitotoxic stress and disease progression across a spectrum of excitotoxicity-associated disorders.

## Materials and methods

### Approval of the study and compliance with ARRIVE guidelines

We have complied with all relevant ethical regulations for animal use. This study was approved by the responsible animal care committee (Regierungspräsidium Karlsruhe, Germany, approval G-102/16). The authors confirm that all experiments were carried out in accordance with German guidelines for the care and use of laboratory animals and with the European Community Council Directive 86/609/EEC. Experimental design and reporting standards comply with ARRIVE guidelines. Biostatistical and biometrical planning was guided by the Department of Medical Biometry at the Institute of Medical Biometry and Informatics at Heidelberg University. Power calculations of animal numbers were done prior to starting the study based on exploratory RT-qPCR data from R6/2 mice (relative expression level of *Bdnf* in WT controls vs R6/2). Sample sizes were calculated using SAS version 9.1 proc power to ensure adequate power with an effect size of 0.8.

### Mice

We used 24–26-week-old heterozygous zQ175 mice^53^, which were kindly provided by Dr. R. Parlato (Medical Faculty Mannheim, Heidelberg University), and their WT littermates. Mice were housed at the interdisciplinary neurobehavioral core facility (INBC; Heidelberg University) on a 12 h light/dark cycle and had ad libitum access to water and food. Each cage (15 × 21 × 13.5 cm) contained one square cotton nestlet as nesting material. After weaning, mice were group-housed (max. four animals/group) until memantine or FP802 treatment started. During treatments, mice were single-housed to ensure correct dosages (see below). One week before sacrifice, mice were transferred to our laboratory (University of Heidelberg, Department of Neurobiology) to prevent novelty-induced gene expression shortly before sacrifice. We compared the relative gene expression level of six genes in two brain regions of one untreated zQ175 group to two treatment groups (memantine, FP802) and to one control group (untreated WT littermates). The tissue of one mouse counted as one experimental unit. 37 mice (15 females and 22 males) were used in this study, and each group contained a mix of both genders. Mice that were heterozygous zQ175 were allocated by simple randomization to the untreated, memantine, and FP802 groups. Health status was checked daily. No adverse events were observed.

### In vivo memantine and compound FP802 treatment

Treatment started 2 months (at age 16–18 weeks) before sacrifice (at age 24–26 weeks). Treatment was performed as described previously^52^. In short, memantine (Sigma-Aldrich) and FP802 (Wuxi AppTec/Chemspace) were dissolved in drinking water. Exact drug concentrations depended on the weight and water intake of each mouse and were calculated to reach a dose of 10 mg/kg (memantine) and 40 mg/kg (FP802) per day. Water consumption was measured daily for the first 2 weeks, and once per week thereafter. Water was replaced three times per week.

### Mouse brain sample preparation

Mice were sacrificed by cervical dislocation; brains were rapidly removed and cut into coronal sections (1 mm) using a brain matrix. Brain sections were stored in RNAlater (Sigma Life Science) for 1 week before microdissecting individual brain regions (motor cortex and striatum) with the help of a binocular microscope (Stemi SV6, Zeiss) under a laminar flow hood. Tissue was stored at −80 °C until total RNA extraction.

### Primary cell culture

Hippocampal neurons from newborn C57Bl/6N (Charles River) mice were prepared, plated (~1.4 × 10^5^ cells/cm^2^; on poly-D-lysine- and laminin-coated (BD Biosciences) plates), and maintained as previously described^40^ with slight modifications. Briefly, until day in vitro (DIV) 8, cells were cultivated in Neurobasal-A (Gibco, #10888022) supplemented with B27 (Gibco, #17504044), 0.5 mM L-glutamine (Sigma-Aldrich, #G7513), 1% rat serum (Biowest, #S2150), and 50 U/ml Penicillin-Streptomycin (PS) (Gibco, #15140122). On DIV 3, 2.8 μM cytosine arabinoside (AraC) (Sigma-Aldrich, #C1768) was added to the culture medium. On DIV 8, medium was changed to neuronal medium containing 10 mM Hepes, pH 7.4; 26.1 mM NaHCO_3_; 114 mM NaCl; 5.3 mM KCl, 2 mM CaCl_2_; 1 mM MgCl_2_; 30 mM glucose; 0.5 mM C_3_H_3_NaO_3_; 1 mM glycine; 0.001% phenol red; 10% phosphate-free Eagle’s minimum essential medium (Gibco; #21090-022); 7.5 μg/ml insulin, 7.5 μg/ml transferrin, 7.5 ng/ml sodium selenite (ITS, Sigma-Aldrich, #I3146); and 50 U/ml PS.

### Assays with primary cells

Experiments were performed on DIV 10 or 11 with cells in 12-well plates (3.8 cm^2^/well, 1 ml medium/well) for RNA isolation and with cells in 3.5 cm dishes (9.6 cm^2^, 2 ml medium/dish) for protein isolation. Drugs were used at the following final concentrations: bicuculline (Bic, 50 µM), tetrodotoxin (TTX, 1 µM), NMDA (30 µM), DL-*threo*-β-Benzyloxyaspartic acid (TBOA, 50 µM), memantine (Mem; 10 µM or 100 µM), MK801 (MK; 10 µM), (2R)-amino-5-phosphonovaleric acid (APV, 50 µM). For RNA isolation, in addition to an untreated control, the following samples were collected (SP indicates “short protocol,” LP indicates “long protocol”). (i) Bic control (induction without interference), 1 h (SP) or 2 h (LP) Bic; (ii) TTX condition (passive effect of excitotoxicity), 0.5 h (SP) or 1 h (LP) Bic + 0.5 h (SP) or 1 h (LP) Bic and TTX; (iii) TTX/NMDA condition (active effect of excitotoxicity), 0.5 h (SP) or 1 h (LP) Bic + 0.5 h (SP) or 1 h (LP) Bic, TTX and NMDA; (iv) TBOA condition (glutamate spillover effect), 1 h Bic + 1 h Bic and TBOA. For protein isolation, in addition to an untreated control, the following samples were collected (“Phos” indicates protocol for phosphorylation analyses, LP indicates “long protocol”). (i) Bic control (induction without interference), 20 min (Phos) or 2 h (LP) Bic; (ii) TTX condition (passive effect of excitotoxicity), 10 min (Phos) or 1 h (LP) Bic + 10 min (Phos) or 1 h (LP) Bic and TTX; (iii) TTX/NMDA condition (active effect of excitotoxicity), 10 min (Phos) or 1 h (LP) Bic + 10 min (Phos) or 1 h (LP) Bic, TTX and NMDA; (iv) NMDA condition (excitotoxicity), 10 min (Phos) or 1 h (LP) Bic + 10 min (Phos) or 1 h (LP) Bic and NMDA.

In the activity-driven gene induction rescue experiment, and in the related death assay, memantine, MK801, and APV were applied 1 h in advance.

For cell death analyses, following the indicated treatments, cells were washed twice with medium, kept in culture for 24 h, and fixed with RotiR-Histofix (Carl Roth GmbH & Co.KG) for 15 min. Nuclei were stained with Hoechst 33258 (Abcam, #ab228550). Cell death was assessed by analyzing the morphology and signal intensity of stained nuclei in 20 visual fields per condition with a fluorescence microscope (40× magnification, Leica DM IRBE). Small and condensed nuclei were considered pyknotic, representing dead cells. Counting was performed semi-automatically using CellProfiler^TM^ and CellProfiler Analyst^TM^ (ver 2.0; Carpenter Lab at Broad Institute).

Schematic representations of the treatment timelines for the collected samples for each experiment are depicted in the respective figures.

### RT-qPCR analyses

Total RNA was isolated using the RNeasy Plus Mini Kit (Qiagen) with on-column DNase I digestion according to the manufacturer’s instructions. For the generation of cDNA, 1 μg of total RNA was reverse transcribed with the High-Capacity cDNA reverse transcription kit (Applied Biosystems). Quantitative RT-qPCR was performed with a StepOnePlus (Applied Biosystems) thermal cycler using TaqMan gene expression assays (Applied Biosystems) for the following genes: *Arc* (Mm00479619_g1), *Atf3* (Mm00476032_m1), *Bdnf* (Mm00432069_m1; transcript containing exon IV), *Fos* (Mm00487425_m1), *Egr1* (Mm00656724_m1), *Homer1* (Mm00516275_m1), *Inhba* (Mm00434338_m1), *Npas4* (Mm00463644_m1), *Nr4a1* (Mm00439358_m1) and *Ppp1r1b* (Mm00454892_m1). Expression levels were normalized to *Gusb* (Mm00446953_m1).

### Immunoblot analyses

Primary cells were lysed in sample buffer (9% SDS, 187.5 mM Tris, 30% glycerol, 10% 2-mercaptoethanol, and bromophenol blue; pH 6.8). Cell extracts were boiled for 5 min at 95 °C and stored at −80 °C. Proteins were separated by 10% SDS-PAGE applying a constant current of 30 mA per gel and transferred to nitrocellulose membranes (Amersham) with a constant voltage of 20 V for 1.5 h. Membranes were blocked with 5% milk (Reform instant skimmed milk powder, Frema) in PBST (0.1% Tween-20 in PBS) at room temperature (RT) for 1 h and incubated with primary antibodies in PBST containing 5% bovine serum albumin overnight at 4 °C. Membranes were washed three times 10 min with PBST, and incubated with secondary antibodies in PBST containing 5% nonfat dried milk at RT for 30 min. Then, membranes were washed again three times 10 min with PBST. Enhanced chemiluminescence detection was performed according to the manufacturer’s instructions (GE Healthcare Life Science). Films were either developed with a Kodak developing machine or with the ChemiDoc^TM^ imaging system (Bio-Rad). Alpha-tubulin signal was used as a loading control for every blot. Signal quantification was performed with Fiji image analysis software (version 2.0.0).

The following primary antibodies were used: anti-phospho-CREB (Ser 133) (Millipore, #06-519); anti-CREB (Cell Signaling, #9197); anti-phospho-Elk1 (B-4) (Ser 383) (Santa Cruz, #sc-8406); anti-Elk1 (E-5) (Santa Cruz, #sc-365876); anti-phospho-ERK1/2 (Thr 202/Tyr 204) (Cell Signaling, #9106); anti-ERK1/2 (Cell Signaling, #9102); anti-total phospho-Ser/Thr (ECM Biosciences, #PP2551); anti-alpha-tubulin (Sigma, #T9026). The following respective secondary antibodies were used: anti-mouse IgG-HRP (Jackson ImmunoResearch, #115-035-003); anti-rabbit IgG-HRP (Jackson ImmunoResearch, #115-035-144).

### Transcriptome analyses

#### RNA sequencing

RNA-seq was performed on DIV11 hippocampal primary cells with 5 biological replicates per treatment. Total RNA was isolated using the RNeasy Plus Mini Kit (Qiagen) with on-column DNase I digestion according to the manufacturer’s instructions. The sequencing library was prepared with the NEBNext Ultra II directional RNA Library Preparation kit with NEBnext Multiplex oligos (New England Biolabs, #E7765). Samples were enriched for poly A RNA with the Poly A module. Single-end sequencing was performed on Illumina NextSeq 500 (Illumina) (75 cycles).

#### RNA-sequencing expression analysis

The raw sequencing reads were mapped using STAR (2.5.4b)^93^ (parameters: --alignIntronMax 100000, --outSJfilterReads Unique, --outSAMmultNmax 1, --outFilterMismatchNoverLmax 0.04) to the mouse genome (Mus Musculus GRCm38) using Ensembl genome annotation (Release 94). Read counting for summarization was done with FeatureCounts (1.6.5; multi-mapping or multi-overlapping reads were not counted, stranded mode was set to “–s 2”)^94^. Differentially expressed genes (DEGs) were determined with R (3.6.3) (http://www.R-project.org/) using the DESeq2 package (1.26.0)^95^. Only genes that had at least one read count in any of the analyzed samples of a particular comparison were subjected to DESeq2. For each gene in each comparison, the *p* value was calculated using the Wald significance test. Resulting *p* values were adjusted for multiple testing using Benjamini–Hochberg correction. Genes with a false discovery rate (FDR) < 0.05 were considered to be differentially expressed. The log2 fold change (L2FC) values were shrunk with the function DESeq2::lfcShrink(type = “normal”) to control for the variance of L2FC estimates for genes with low read counts. For a concise data structure and overview, the results of the 14 DEG analyses were merged into one table reflecting a union of the analysis-ready genes per DEG analysis (32,438 genes) (Supplementary Table 1A).

Genes with significant changes over the series of Bic treatments (controls, “0.5 h Bic,” “1 h Bic,” and “2 h Bic”) were determined as an optional criterion for candidate selection. For this, size-factor normalized counts were subjected to an ANOVA per gene. Multiple testing was accounted for on the level of FDR < 0.05 (Supplementary Table 1A).

#### Clustering and categorization

A principal component analysis (PCA) was conducted on the “regularized log” transformed expression values of the 500 most varying genes over all samples using the function DESeq2::plotPCA().

For categorization of genes according to Bic activity classes, the mean of size-factor normalized counts per treatment group was calculated, and selection rules were applied to generate the classes (Supplementary Table 1B).

Shut-off effects (i.e., relative downregulation of upregulated genes) were categorized according to a passive component, determined from the comparison of Bic + Bic/TTX treatment versus Bic-only treatment, or according to an active component, as determined from the comparison of Bic + Bic/TTX/NMDA treatment versus Bic + Bic/TTX. Analogously, a TBOA shut-off was determined from the comparison of Bic + Bic/TBOA treatment versus Bic-only treatment. In addition to the implicit selector L2FC < 0 on the pair-wise comparisons, an FDR < 0.05 was used as a significance threshold (Supplementary Table 1C).

A t-SNE analysis was done for gene candidates fulfilling the following criteria: (1) expression change upon Bic treatment (ANOVA FDR < 0.01), (2) classified to a Bic activity class, and (3) an L2FC value assigned by DESeq2 in all 14 pair-wise comparisons. t-SNE analysis was performed using the R package Rtsne (parameters: check_duplicates = FALSE, theta = 0) after setting a seed for reproducibility via the R function set.seed(102).

Gene-wise similarities of AP firing interference effects were calculated using the equation *s* = *a*·*b*/sqrt(*a*^2^ + *b*^2^), where *a* and *b* are L2FC values of the compared interference conditions “Total excitotoxicity” (TTX/NMDA vs control) and “Glutamate spillover” (TBOA vs control), respectively. Pair-wise Pearson correlation between selected interference conditions was calculated based on the L2FC values of the 569 genes of the activity class “late-induced” with the R function cor().

#### Gene ontology analysis

GO term overrepresentation was analyzed among AP firing-upregulated genes belonging to the passive shut-off (Bic + Bic/TTX vs Bic) category or to the active shut-off (Bic + Bic/TTX vs Bic + Bic/TTX/NMDA) category with the g:GOSt function of g:Profiler^96^ using all expressed genes (DEG analysis adjusted *p* available for all comparisons of Bic treatment time points and the untreated control) as the effective genomic background and Benjamini–Hochberg FDR as the multiple testing correction method. For comparing the clusters of genes in the passive shut-off, the active shut-off, and the shut-off resistant (the remaining upregulated genes) categories, compareCluster of the R package clusterProfiler^97^ was used with the *p* value cutoff of 0.01 and *q* value cutoff of 0.05. All expressed genes formed the effective background, and Benjamini–Hochberg FDR was used for multiple testing correction.

### Statistics and reproducibility

Statistical analyses were conducted in Prism^TM^ 6.0 (GraphPad) or R (R Core Team). The normality of each data set was tested with the Shapiro-Wilk test. Hypothesis tests, number of replicates, and significances are indicated in figure legends. For in vitro experiments, each primary neuronal culture preparation was defined as one replicate; for in vivo experiments, each mouse was defined as one replicate. Two samples from untreated zQ175 were excluded a posteriori from RT-qPCR data since they were identified as outliers with the robust regression and outlier removal test (ROUT, *Q* = 0.5%). They were excluded for all analyzed genes and regions. No other data was excluded. For in vivo gene expression experiments, the experimenter was blinded to group allocation from brain dissection until RT-qPCR data normalization to WT controls.

### Reporting summary

Further information on research design is available in the Nature Portfolio Reporting Summary linked to this article.

## Supplementary information

Supplementary Information

Description of Additional Supplementary Files

Supplementary Data 1

Supplementary Data 2

Reporting Summary

## Data Availability

RNA-Seq data discussed in this publication have been deposited in NCBI’s Gene Expression Omnibus and are accessible through GEO Series accession number GSE184681. Other source data are available in Supplementary Data 2. Descriptive statistics for animal experiments is in Supplementary Tables 2A and 2B in Supplementary Information, and uncropped and unedited blot images are also available in Supplementary Information.
