# Supplementary material for: Inhba, Homer1 and Bdnf are major targets of transcriptomic dysregulation by neurodegenerative disease-associated excitotoxic NMDA receptor signaling
Source: Commun Biol. 2025 Dec 3;8:1743. doi: 10.1038/s42003-025-09074-9 (PMC12678830; doi:10.1038/s42003-025-09074-9)

Supplementary Table 1A: DEG overview

internal comparison name	"treatment" group	base group	genes anayzed*	total DEGs**	up-regulated DEGs in "treatment" group	down-regulated DEGs in "treatment" group
Bic1vs05	1.0h Bic	0.5h Bic	26,859	207	193	14
Bic2vs1	2.0h Bic	1.0h Bic	27,614	2,004	902	1,102
CTRL05B	0.5h Bic	control	26,724	84	83	1
CTRL1B	1.0h Bic	control	27,382	409	313	96
CTRL2B	2.0h Bic	control	27,515	3,202	1,518	1,684
1hBTTX	0.5h Bic + 0.5h TTX	1h Bic	28,366	82	8	74
1hBTTXNMDA	0.5h Bic + 0.5h TTX & NMDA	0.5h Bic + 0.5h TTX	28,732	1,927	456	1,471
1hBTTXNMDAvs1hB	0.5h Bic + 0.5h TTX & NMDA	1h Bic	28,003	1,898	438	1,460
2hBTTX	1h Bic + 1h TTX	2h Bic	28,529	2,347	1,301	1,046
2hBTTXNMDA	1h Bic + 1h TTX & NMDA	1h Bic + 1h TTX	29,477	7,768	3,758	4,010
2hBTTXNMDAvs2hB	1h Bic + 1h TTX & NMDA	2h Bic	28,791	7,284	3,480	3,804
2hBTBOA	1h Bic + 1h TBOA	1h Bic + 1h TTX	29,101	1,558	350	1,208
2hBTBOAvs2hB	1h Bic + 1h TBOA	2h Bic	28,305	310	36	274
2hBTBOA_TTXNMDA	1h Bic + 1h TBOA	1h Bic + 1h TTX & NMDA	29,275	5,485	2,971	2,514

* all genes that show at least one read over all samples of the compared groups

** DEGs are defined to have an adjusted p-value below 0.05

__Supplementary Table 1B: Bic Activity classes__

32,438 genes were subjected to the following classification.

Activity class	Classification rule	Activity class		Number of genes	alysis (Fig.3D-F + Supl. Fig.1)
Late upregulated	padj.CTRL2B <= 0.05 **&** mean.05hBic < mean.1hbic **&** mean.1hbic <= mean.2hbic	late.induced		1,267	569
Early upregulated	padj.CTRL1B <= 0.05 **&** mean.05hBic < mean.1hbic **&** mean.1hbic >= mean.2hbic	early.induced		45	28
No activity	padj.CTRL05B > 0.05 **&** padj.CTRL1B > 0.05 **&** padj.CTRL2B > 0.05	not.active		14,155	-
Late downregulated	padj.CTRL2B <= 0.05 **&** mean.05hBic > mean.1hbic **&** mean.1hbic >= mean.2hbic	late.downregulated		1,554	717
Early downregulated	padj.CTRL1B <= 0.05 **&** mean.05hBic > mean.1hbic **&** mean.1hbic <= mean.2hbic	early.upregulated		11	1
Remaining	"none of the rules above apply"	none		15,406	-
			Total	*32,438*	*1,315*

Supplementary Table 1C: Shut Off classes

32,438 genes were subjected to the follwing classification.

Shut Off class	Classification rule	Number of genes
**Late Bic upregulated genes**		
Total late upregulated	activity class = late upregulated **&** padj.2hBTTX, l2fc. 2hBTTX, padj.2hBTTXNMDA, l2fc.2hBTTXNMDA is not empty	1,137
TTX no, NMDA no	Total late upregulated **&** padj.2hBTTX > 0.05 **&** padj.2hBTTXNMDA > 0.05	282
TTX no, NMDA down	Total late upregulated **&** padj.2hBTTX > 0.05 **&** padj.2hBTTXNMDA < 0.05 **&** l2fc.2hBTTXNMDA < 0	393
TTX no, NMDA up	Total late upregulated **&** padj.2hBTTX > 0.05 **&** padj.2hBTTXNMDA < 0.05 **&** l2fc.2hBTTXNMDA > 0	73
TTX up, NMDA no	Total late upregulated **&** padj.2hBTTX < 0.05 **&** l2fc.2hBTTX > 0 **&** padj.2hBTTXNMDA > 0.05	3
TTX up, NMDA down	Total late upregulated **&** padj.2hBTTX < 0.05 **&** l2fc.2hBTTX > 0 **&** padj.2hBTTXNMDA < 0.05 **&** l2fc.2hBTTXNMDA < 0	57
TTX up, NMDA up	Total late upregulated **&** padj.2hBTTX < 0.05 **&** l2fc.2hBTTX > 0 **&** padj.2hBTTXNMDA < 0.05 **&** l2fc.2hBTTXNMDA > 0	-
TTX down, NMDA no	Total late upregulated **&** padj.2hBTTX < 0.05 **&** l2fc.2hBTTX < 0 **&** padj.2hBTTXNMDA > 0.05	149
TTX down, NMDA down	Total late upregulated **&** padj.2hBTTX < 0.05 **&** l2fc.2hBTTX < 0 **&** padj.2hBTTXNMDA < 0.05 **&** l2fc.2hBTTXNMDA < 0	124
TTX down, NMDA up	Total late upregulated **&** padj.2hBTTX < 0.05 **&** l2fc.2hBTTX < 0 **&** padj.2hBTTXNMDA < 0.05 **&** l2fc.2hBTTXNMDA > 0	56
TTX down = total passive shut off	activity class = late upregulated **&** padj.2hBTTX < 0.05 **&** l2fc.2hBTTX < 0	329
NMDA down = total active shut of	activity class = late upregulated **&** padj.2hBTTXNMDA < 0.05 **&** l2fc.2hBTTXNMDA < 0	574
TBOA down = total TBOA shut off	activity class = late upregulated **&** padj.2hBTBOA< 0.05 **&** l2fc.2hBTBOA < 0	192
**Early Bic upregulated genes**		
Total early upregulated	activity class = early upregulated **&** padj.1hBTTX, l2fc. 1hBTTX, padj.1hBTTXNMDA, l2fc.1hBTTXNMDA is not empty	35
TTX no, NMDA no	Total early upregulated **&** padj.1hBTTX > 0.05 **&** padj.1hBTTXNMDA > 0.05	13
TTX no, NMDA down	Total early upregulated **&** padj.1hBTTX > 0.05 **&** padj.1hBTTXNMDA <= 0.05 **&** l1fc.1hBTTXNMDA < 0	6
TTX no, NMDA up	Total early upregulated **&** padj.1hBTTX > 0.05 **&** padj.1hBTTXNMDA <= 0.05 **&** l1fc.1hBTTXNMDA > 0	1
TTX up, NMDA no	Total early upregulated **&** padj.1hBTTX < 0.05 **&** l1fc.1hBTTX > 0 **&** padj.1hBTTXNMDA > 0.05	-
TTX up, NMDA down	Total early upregulated **&** padj.1hBTTX < 0.05 **&** l1fc.1hBTTX > 0 **&** padj.1hBTTXNMDA < 0.05 **&** l1fc.1hBTTXNMDA < 0	-
TTX up, NMDA up	Total early upregulated **&** padj.1hBTTX < 0.05 **&** l1fc.1hBTTX > 0 **&** padj.1hBTTXNMDA < 0.05 **&** l1fc.1hBTTXNMDA > 0	-
TTX down, NMDA no	Total early upregulated **&** padj.1hBTTX < 0.05 & l1fc.1hBTTX < 0 **&** padj.1hBTTXNMDA > 0.05	5
TTX down, NMDA down	Total early upregulated **&** padj.1hBTTX < 0.05 **&** l1fc.1hBTTX < 0 **&** padj.1hBTTXNMDA <= 0.05 **&** l1fc.1hBTTXNMDA < 0	5
TTX down, NMDA up	Total early upregulated **&** padj.1hBTTX < 0.05 **&** l1fc.1hBTTX < 0 **&** padj.1hBTTXNMDA < 0.05 **&** l1fc.1hBTTXNMDA > 0	5
TTX down = total passive shut off	activity class = early upregulated **&** padj.1hBTTX < 0.05 **&** l1fc.1hBTTX < 0	15
NMDA down = total active shut of	activity class = early upregulated **&** padj.1hBTTXNMDA < 0.05 **&** l1fc.1hBTTXNMDA < 0	11

Supplementary Table 2A (Fig. 6B; Motor Cortex)

Inhba	WT	zQ175	zQ175 + Mem	zQ175 + FP802
Mean	1	0.9572	0.9766	1.056
Std. Deviation	0.1092	0.1258	0.2671	0.5119
Std. Error of Mean	0.03153	0.04192	0.1009	0.2289
Ordinary one-way ANOVA with Tukey R squared	0.01918			

Tukey's multiple comparisons t	WT vs. zQ175	WT vs. zQ175 + Mem	WT vs. zQ175 + FP802	zQ175 vs. zQ175 + Mem	zQ175 vs. zQ175 + FP802	zQ175 + Mem vs. zQ175 + FP802
Mean diff.	0.04282	0.02345	-0.05613	-0.01937	-0.09894	-0.07957
95.00% CI of diff.	-0.2509 to 0.3366	-0.2934 to 0.3403	-0.4107 to 0.2985	-0.3551 to 0.3164	-0.4705 to 0.2726	-0.4697 to 0.3105

Bdnf	WT	zQ175	zQ175 + Mem	zQ175 + FP802
Mean	1	1.027	1.211	1.307
Std. Deviation	0.16	0.2514	0.4032	0.553
Std. Error of Mean	0.04619	0.08381	0.1524	0.2473
Ordinary one-way ANOVA with Tukey R squared	0.1357			

Tukey's multiple comparisons t	WT vs. zQ175	WT vs. zQ175 + Mem	WT vs. zQ175 + FP802	zQ175 vs. zQ175 + Mem	zQ175 vs. zQ175 + FP802	zQ175 + Mem vs. zQ175 + FP802
Mean diff.	-0.02696	-0.2112	-0.3071	-0.1843	-0.2801	-0.09586
95.00% CI of diff.	-0.4125 to 0.3586	-0.6270 to 0.2046	-0.7724 to 0.1583	-0.6248 to 0.2563	-0.7678 to 0.2075	-0.6078 to 0.4161

Homer1	WT	zQ175	zQ175 + Mem	zQ175 + FP802
Mean	1	0.8129	0.8946	1.011
Std. Deviation	0	0.02054	0.2012	0.2823
Std. Error of Mean	0	0.01453	0.07604	0.1262
Ordinary one-way ANOVA with Tukey R squared	0.1329			

Tukey's multiple comparisons t	WT vs. zQ175	WT vs. zQ175 + Mem	WT vs. zQ175 + FP802	zQ175 vs. zQ175 + Mem	zQ175 vs. zQ175 + FP802	zQ175 + Mem vs. zQ175 + FP802
Mean diff.	0.1871	0.1054	-0.01082	-0.08169	-0.1979	-0.1162
95.00% CI of diff.	-0.3172 to 0.6914	-0.2596 to 0.4704	-0.4015 to 0.3798	-0.5486 to 0.3852	-0.6851 to 0.2893	-0.4572 to 0.2248

Nr4a1	WT	zQ175	zQ175 + Mem	zQ175 + FP802
Mean	0.9756	1.142	2.219	2.886
Std. Deviation	0.3693	0.6961	1.142	1.534
Std. Error of Mean	0.1066	0.232	0.4316	0.6861
Ordinary one-way ANOVA with Tukey R squared	0.4361			

Tukey's multiple comparisons t	WT vs. zQ175	WT vs. zQ175 + Mem	WT vs. zQ175 + FP802	zQ175 vs. zQ175 + Mem	zQ175 vs. zQ175 + FP802	zQ175 + Mem vs. zQ175 + FP802
Mean diff.	-0.1661	-1.243	-1.91	-1.077	-1.744	-0.6669
95.00% CI of diff.	-1.227 to 0.8948	-2.387 to -0.09881	-3.191 to -0.6293	-2.289 to 0.1356	-3.086 to -0.4018	-2.076 to 0.7420

Npas4	WT	zQ175	zQ175 + Mem	zQ175 + FP802
Mean	1.041	1.153	2.565	2.077
Std. Deviation	0.2861	0.5225	2.975	3.015
Std. Error of Mean	0.08258	0.1742	1.125	1.348
Ordinary one-way ANOVA with Tukey R squared	0.1243			

Tukey's multiple comparisons t	WT vs. zQ175	WT vs. zQ175 + Mem	WT vs. zQ175 + FP802	zQ175 vs. zQ175 + Mem	zQ175 vs. zQ175 + FP802	zQ175 + Mem vs. zQ175 + FP802
Mean diff.	-0.1127	-1.525	-1.036	-1.412	-0.9238	0.4882
95.00% CI of diff.	-2.259 to 2.034	-3.840 to 0.7903	-3.627 to 1.555	-3.865 to 1.041	-3.639 to 1.791	-2.362 to 3.338

Supplementary Table 2B (Fig. 6C; Striatum)

Pp1r1b	WT	zQ175	zQ175 + Mem	zQ175 + FP802
Mean	0.9856	0.5842	0.7868	0.8981
Std. Deviation	0.1215	0.07242	0.2044	0.422
Std. Error of Mean	0.03664	0.02414	0.07725	0.1887
Ordinary one-way ANOVA with Tukey R squared	0.4215			

Tukey's multiple comparisons test	WT vs. zQ175	WT vs. zQ175 + Mem	WT vs. zQ175 + FP802	zQ175 vs. zQ175 + Mem	zQ175 vs. zQ175 + FP802	zQ175 + Mem vs. zQ175 + FP802
Mean diff.	0.4014	0.1988	0.08752	-0.2027	-0.3139	-0.1113
95.00% CI of diff.	0.1525 to 0.6504	-0.06905 to 0.4666	-0.2113 to 0.3863	-0.4818 to 0.07650	-0.6229 to -0.004953	-0.4356 to 0.2131

Inhba	WT	zQ175	zQ175 + Mem	zQ175 + FP802
Mean	1	0.6637	0.8073	0.9803
Std. Deviation	0.08784	0.1086	0.2265	0.3583
Std. Error of Mean	0.02536	0.03621	0.08562	0.1463
Ordinary one-way ANOVA with Tukey R squared	0.378			

Tukey's multiple comparisons test	WT vs. zQ175	WT vs. zQ175 + Mem	WT vs. zQ175 + FP802	zQ175 vs. zQ175 + Mem	zQ175 vs. zQ175 + FP802	zQ175 + Mem vs. zQ175 + FP802
Mean diff.	0.3363	0.1927	0.01969	-0.1436	-0.3166	-0.173
95.00% CI of diff.	0.1037 to 0.5689	-0.05821 to 0.4435	-0.2441 to 0.2834	-0.4095 to 0.1222	-0.5946 to -0.03858	-0.4665 to 0.1205

Bdnf	WT	zQ175	zQ175 + Mem	zQ175 + FP802
Mean	1	0.5511	1.627	1.712
Std. Deviation	0.4208	0.2567	0.8652	1.119
Std. Error of Mean	0.1215	0.08555	0.327	0.5005
Ordinary one-way ANOVA with Tukey R squared	0.3566			

Tukey's multiple comparisons test	WT vs. zQ175	WT vs. zQ175 + Mem	WT vs. zQ175 + FP802	zQ175 vs. zQ175 + Mem	zQ175 vs. zQ175 + FP802	zQ175 + Mem vs. zQ175 + FP802
Mean diff.	0.4489	-0.6266	-0.7124	-1.076	-1.161	-0.08574
95.00% CI of diff.	-0.3231 to 1.221	-1.459 to 0.2061	-1.644 to 0.2196	-1.958 to -0.1932	-2.138 to -0.1847	-1.111 to 0.9394

Homer1	WT	zQ175	zQ175 + Mem	zQ175 + FP802
Mean	0.9539	0.5575	0.7703	0.9586
Std. Deviation	0.1106	0.05712	0.2047	0.2582
Std. Error of Mean	0.03334	0.01904	0.07736	0.1155
Ordinary one-way ANOVA with Tukey R squared	0.5804			

Tukey's multiple comparisons test	WT vs. zQ175	WT vs. zQ175 + Mem	WT vs. zQ175 + FP802	zQ175 vs. zQ175 + Mem	zQ175 vs. zQ175 + FP802	zQ175 + Mem vs. zQ175 + FP802
Mean diff.	0.3965	0.1836	-0.004639	-0.2129	-0.4011	-0.1882
95.00% CI of diff.	0.2072 to 0.5858	-0.02005 to 0.3872	-0.2318 to 0.2225	-0.4251 to -0.0006097	-0.6360 to -0.1662	-0.4349 to 0.05839

Nr4a1	WT	zQ175	zQ175 + Mem	zQ175 + FP802
Mean	1	0.7571	2.236	3.819
Std. Deviation	0.529	0.6164	1.62	2.34
Std. Error of Mean	0.1527	0.2055	0.6122	1.047
Ordinary one-way ANOVA with Tukey R squared	0.4658			

Tukey's multiple comparisons test	WT vs. zQ175	WT vs. zQ175 + Mem	WT vs. zQ175 + FP802	zQ175 vs. zQ175 + Mem	zQ175 vs. zQ175 + FP802	zQ175 + Mem vs. zQ175 + FP802
Mean diff.	0.2429	-1.236	-2.819	-1.479	-3.062	-1.583
95.00% CI of diff.	-1.233 to 1.719	-2.828 to 0.3559	-4.601 to -1.038	-3.165 to 0.2079	-4.929 to -1.195	-3.543 to 0.3763

Npas4	WT	zQ175	zQ175 + Mem	zQ175 + FP802
Mean	1	0.7326	1.486	1.308
Std. Deviation	0.3957	0.5811	1.77	1.298
Std. Error of Mean	0.1142	0.1937	0.6258	0.6489
Ordinary one-way ANOVA with Tukey R squared	0.07894			

Tukey's multiple comparisons test	WT vs. zQ175	WT vs. zQ175 + Mem	WT vs. zQ175 + FP802	zQ175 vs. zQ175 + Mem	zQ175 vs. zQ175 + FP802	zQ175 + Mem vs. zQ175 + FP802
Mean diff.	0.2674	-0.4865	-0.3076	-0.7538	-0.5749	0.1789
95.00% CI of diff.	-0.9828 to 1.518	-1.781 to 0.8077	-1.944 to 1.329	-2.132 to 0.6239	-2.279 to 1.129	-1.557 to 1.915

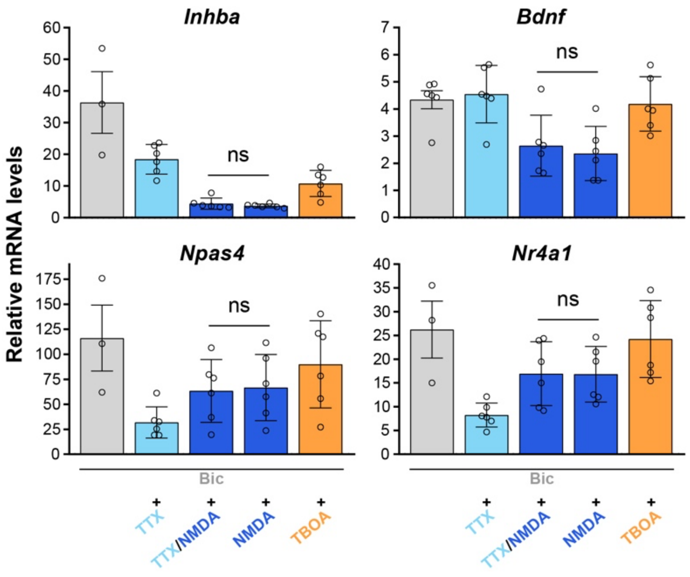

Supplemental Figure 1. The effects of action potential (AP) firing interference with TTX/NMDA or NMDA are highly similar. RT-qPCR quantifications of passive (TTX; i.e., AP firing cessation) and active (TTX/NMDA or NMDA; i.e., NMDAR signaling) effects of esNMDAR signaling-evoked transcriptional dysregulation, and of the effect of TBOA-evoked glutamate spillover (TBOA; i.e., glutamate transporter block). Relative gene expression after LP (see Fig. 1D) was measured and the results are presented as fold change of the mRNA levels compared to control condition. *n* = 3-6. Mean ± SEM; ns = not significant; one-way ANOVAs with Tukey's test.

Fig. 4A: P-CREB and CREB, short protocol (10 min + 10 min), n=6
Sample order always from left to right.
1 = untreated Ctrl
2 = 20 min Bic
3 = 10 min Bic + 10 min Bic/TTX
4 = 10 min Bic + 10 min Bic/TTX/NMDA
5 = 10 min Bic + 10 min Bic/NMDA

Experiment 2:
Tubulin (upper band) and P-CREB (lower band)

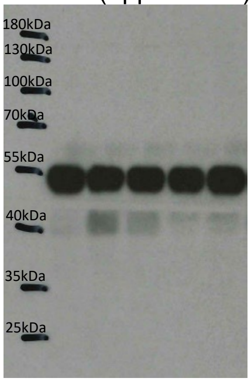

Tubulin (upper band) and CREB (lower band)

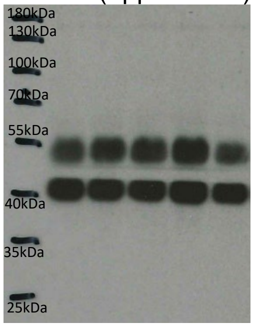

Experiment 4:
P-CREB

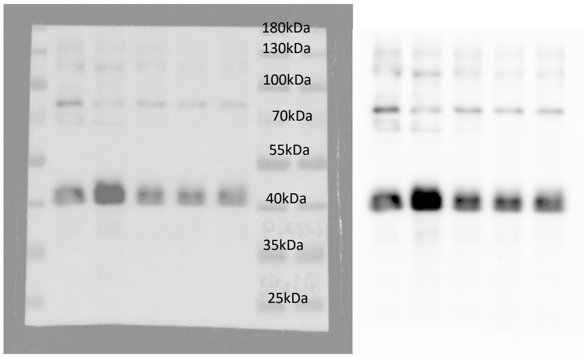

Tubulin

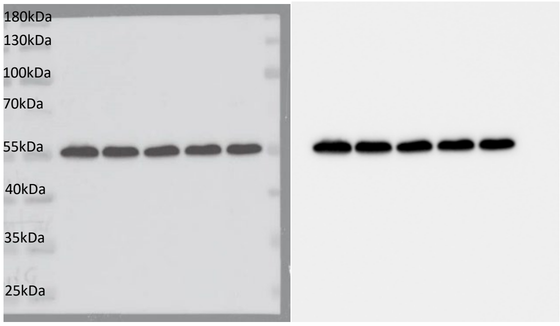

CREB

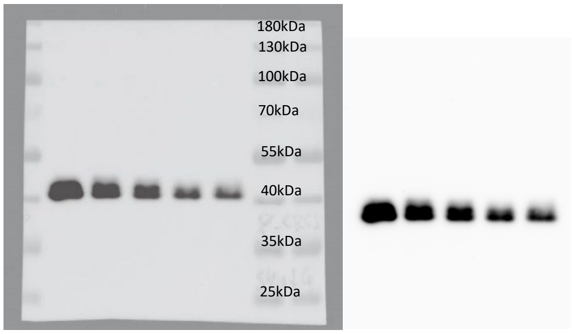

Tubulin

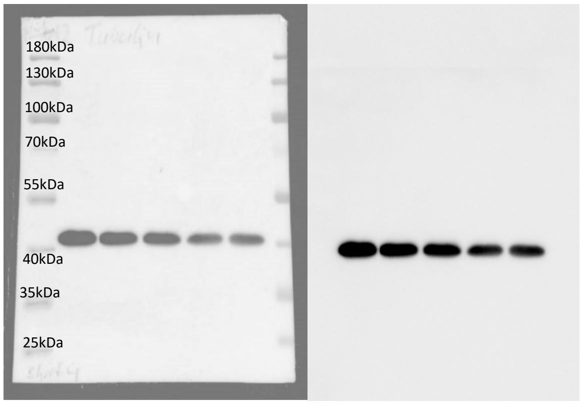

Experiment 5:

P-CREB

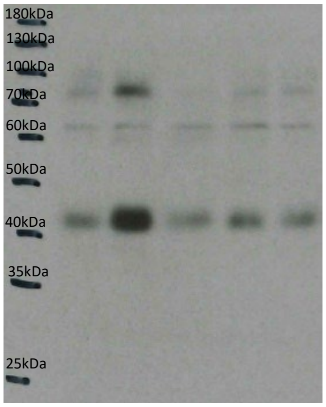

CREB

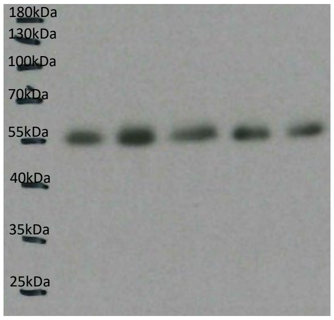

Tubulin

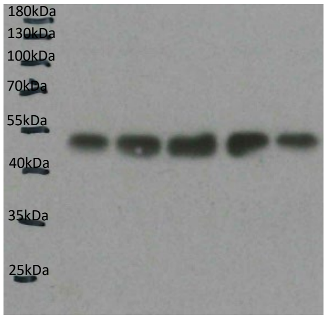

Experiment 6:

P-CREB

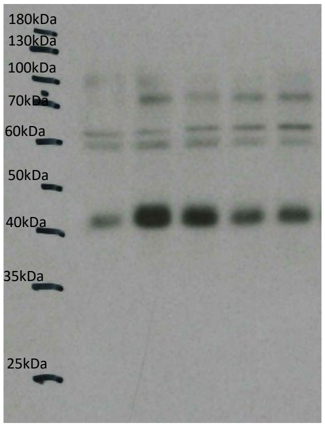

CREB

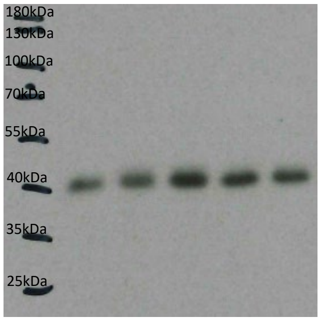

Tubulin

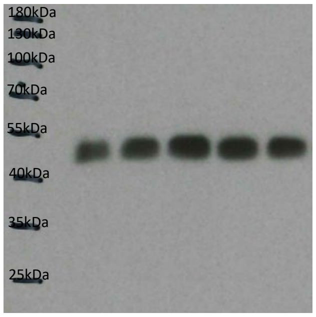

Experiment 7:

P-CREB

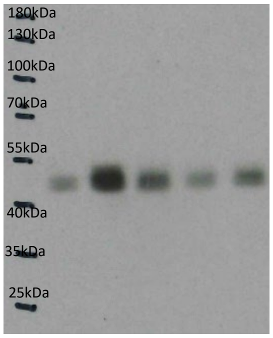

CREB

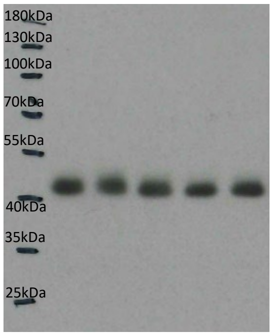

Tubulin

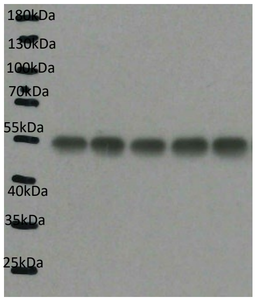

Experiment 8:

P-CREB

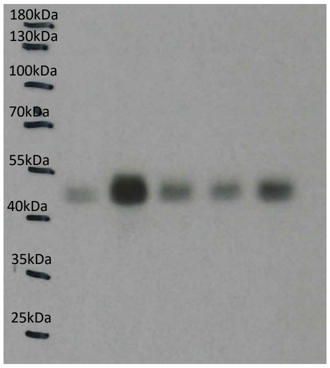

CREB

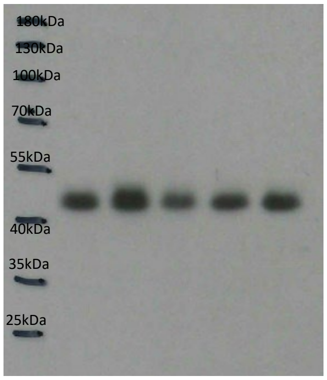

Tubulin

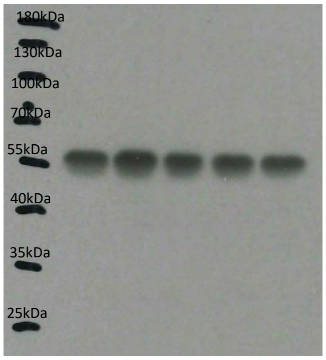

Fig. 4B: P-ERK1/2 and ERK1/2, short protocol (10 min + 10 min), n=6

Sample order always from left to right.

1 = untreated Ctrl

2 = 20 min Bic

3 = 10 min Bic + 10 min Bic/TTX

4 = 10 min Bic + 10 min Bic/TTX/NMDA

5 = 10 min Bic + 10 min Bic/NMDA

Experiment 6:

P-ERK1/2

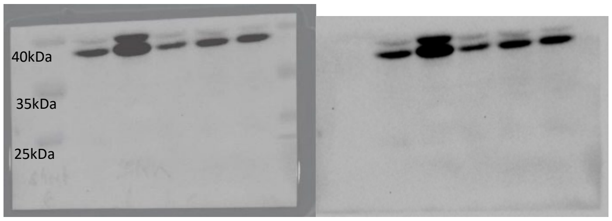

ERK1/2

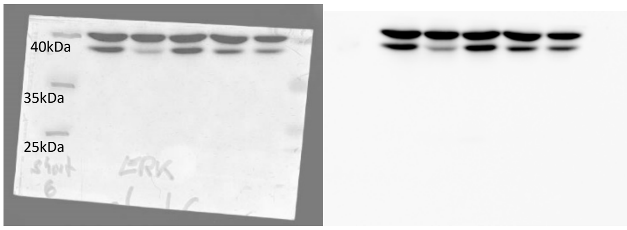

Tubulin

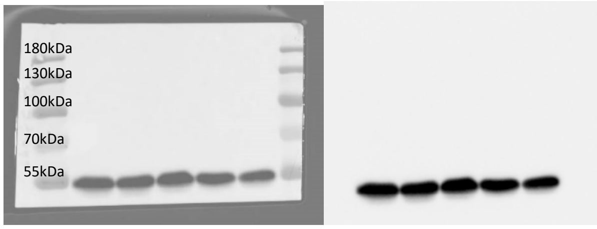

Experiment 7:

P-ERK1/2

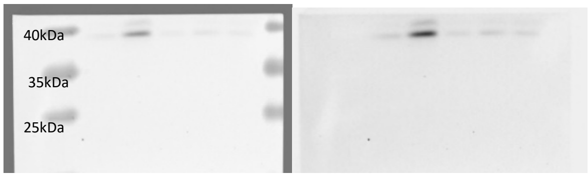

ERK1/2

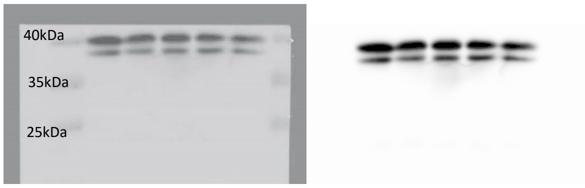

Tubulin

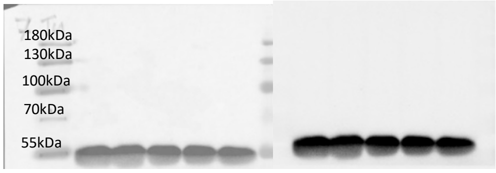

Experiment 2:
P-ERK1/2; ERK1/2; Tubulin

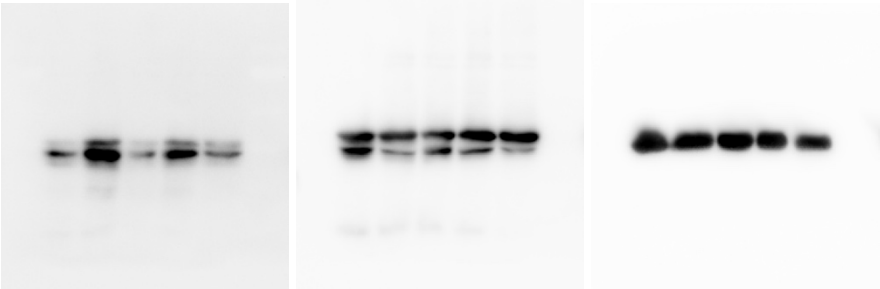

Experiment 3:
P-ERK1/2; ERK1/2; Tubulin

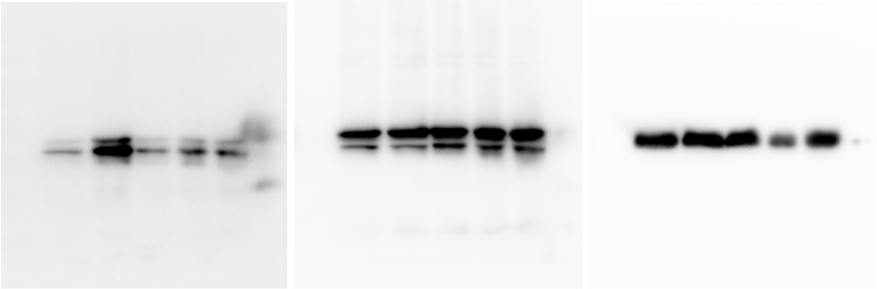

Experiment 4: P-ERK1/2; ERK1/2; Tubulin

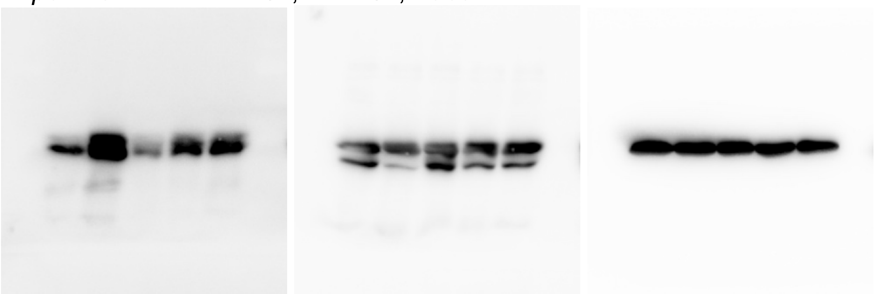

Experiment 8: P-ERK1/2; ERK1/2; Tubulin

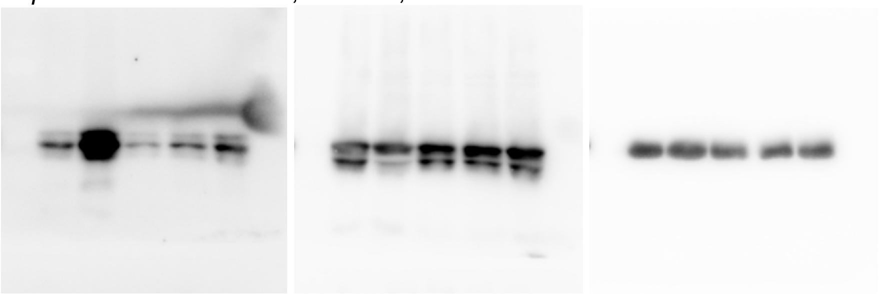

Composite image; experiments 4, 8, 2, 3; protein size markers + P-ERK1/2

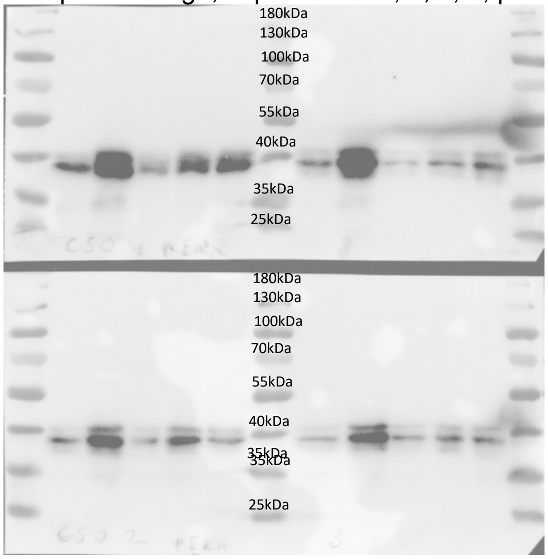

Composite image; experiments 4, 8, 2, 3; protein size markers + ERK1/2

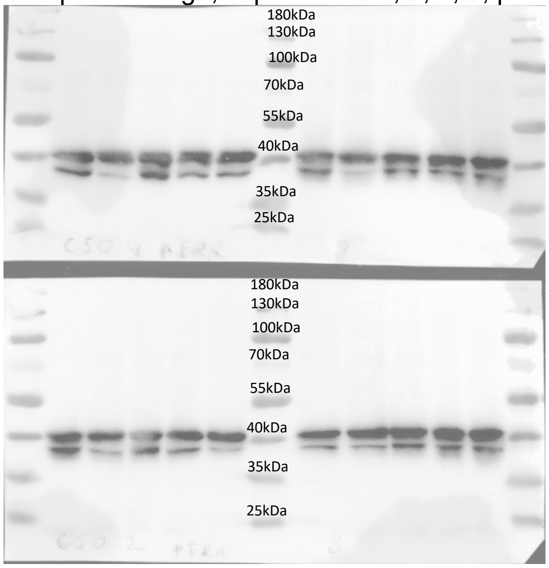

Composite image; experiments 4, 8, 2, 3; protein size markers + Tubulin

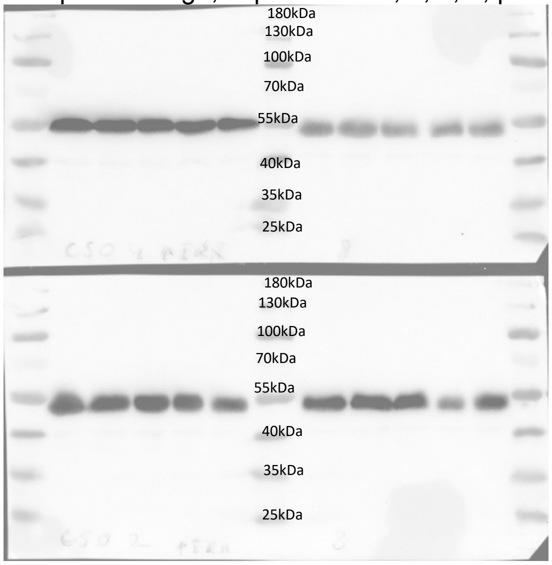

Fig. 4C: P-ELK1 and ELK1, short protocol (10 min + 10 min), n=4

Sample order always from left to right.

1 = untreated Ctrl

2 = 20 min Bic

3 = 10 min Bic + 10 min Bic/TTX

4 = 10 min Bic + 10 min Bic/TTX/NMDA

5 = 10 min Bic + 10 min Bic/NMDA

Experiment 3:

P-ELK1; ELK1

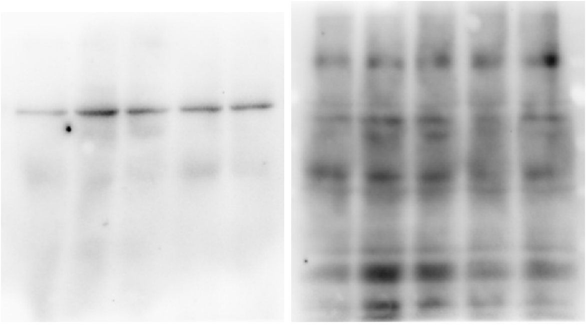

= ELK1

Tubulin (P-ELK1 blot); Tubulin (ELK1 blot)

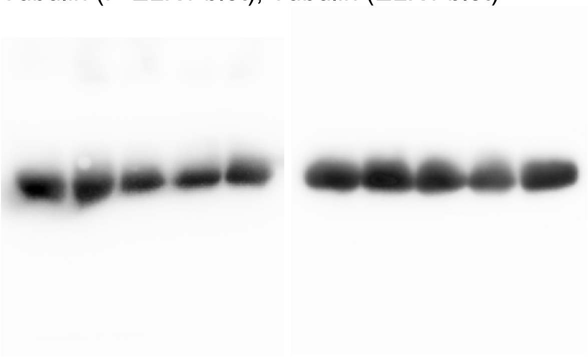

Experiment 4:
P-ELK1; ELK1

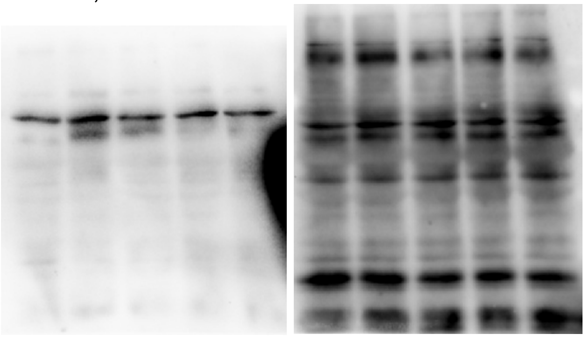

= ELK1

Tubulin (P-ELK1 blot); Tubulin (ELK1 blot)

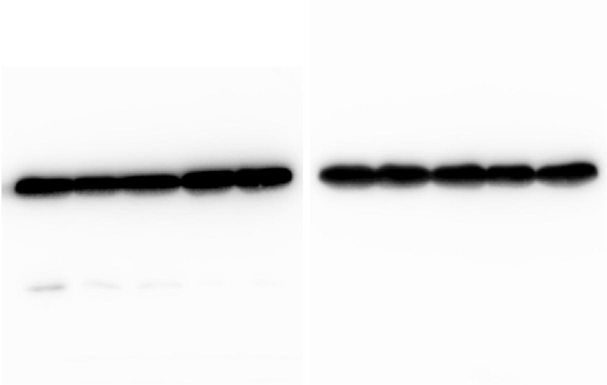

Experiment 7:
P-ELK1; ELK1

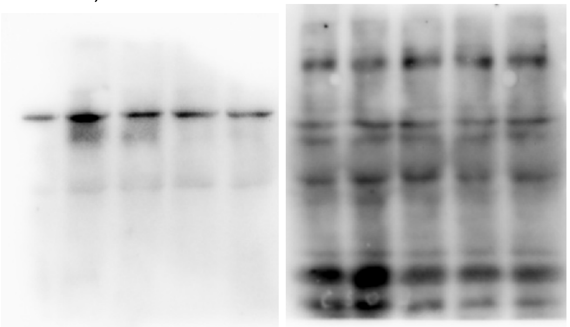

= ELK1

Tubulin (P-ELK1 blot); Tubulin (ELK1 blot)

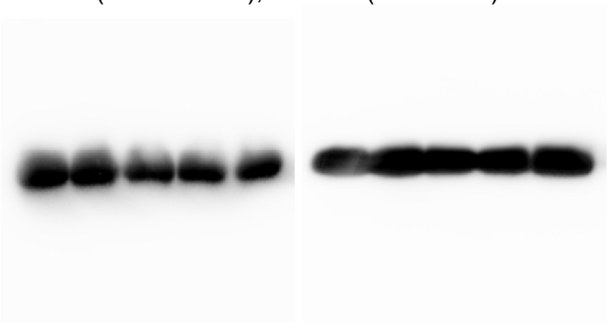

Experiment 8:
P-ELK1; ELK1

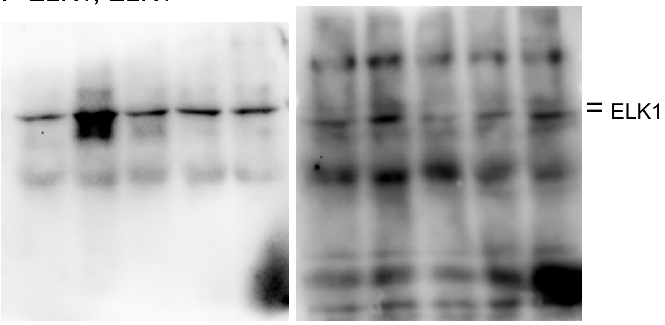

Tubulin (P-ELK1 blot); Tubulin (ELK1 blot)

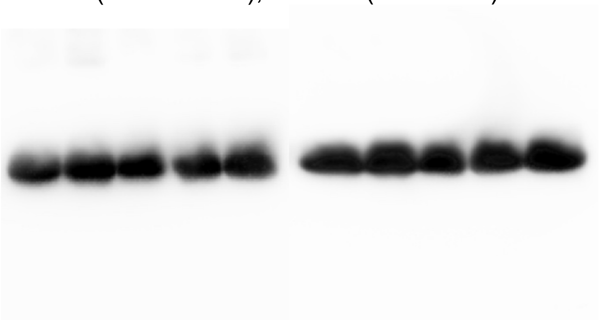

Composite image; experiments 3, 4, 7, 8; protein size markers + P-ELK1

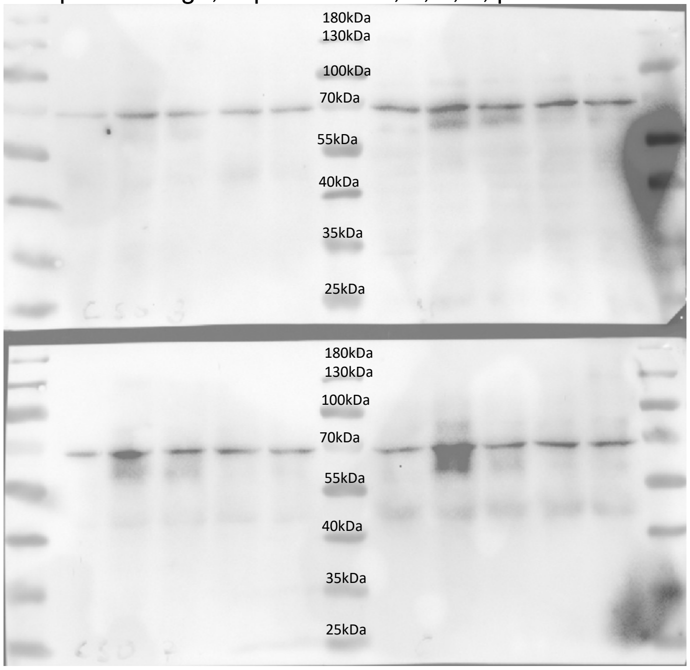

Composite image; experiments 3, 4, 7, 8; protein size markers + ELK1

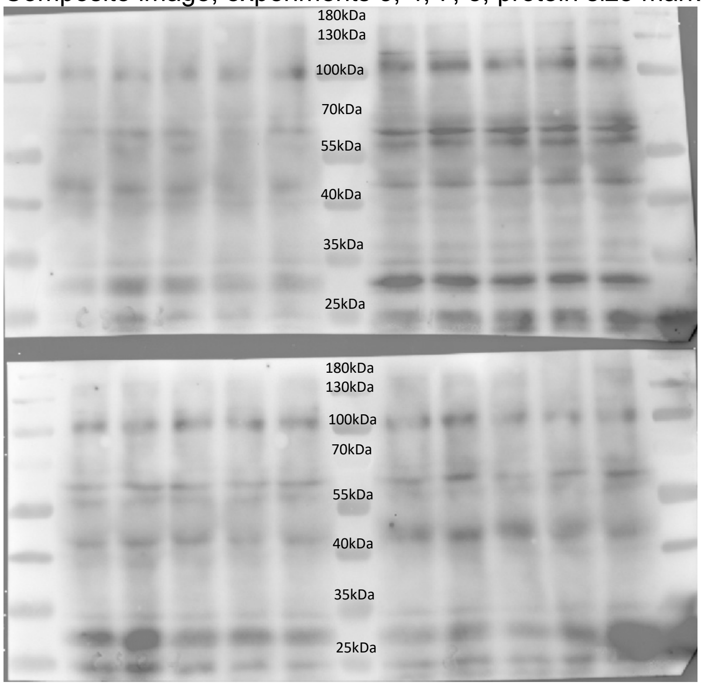

Composite image; experiments 3, 4, 7, 8; protein size markers + Tubulin

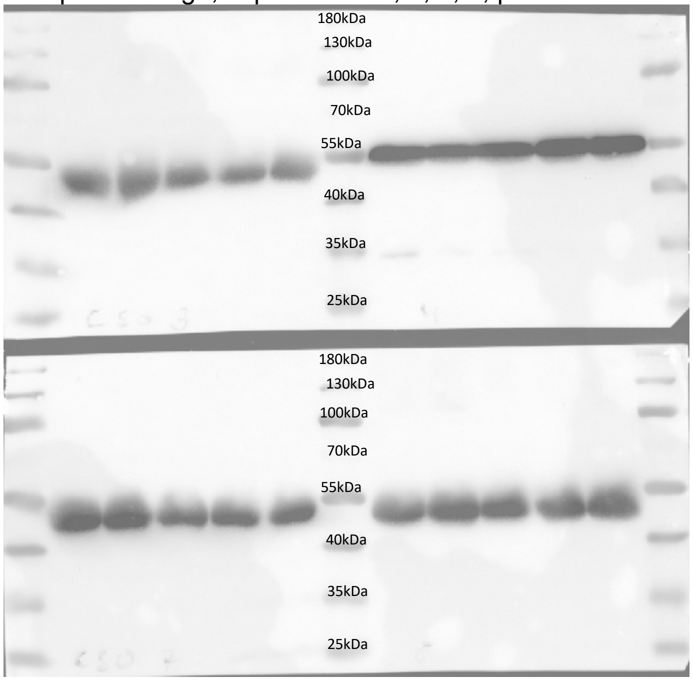

Fig. 4F: CREB, long protocol (1 h + 1 h), n=4
Sample order always from left to right.
1 = untreated Ctrl
2 = 20 min Bic
3 = 10 min Bic + 10 min Bic/TTX
4 = 10 min Bic + 10 min Bic/TTX/NMDA
5 = 10 min Bic + 10 min Bic/NMDA

Experiment 1:
CREB

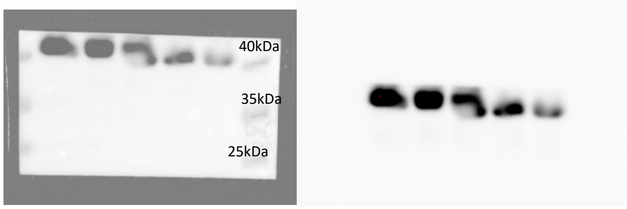

Tubulin

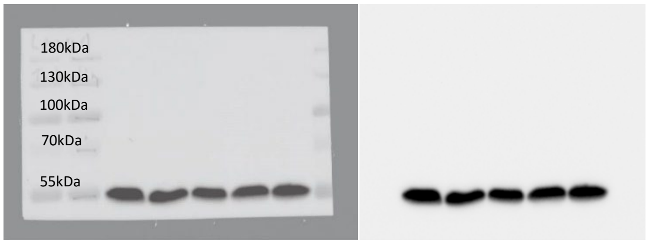

Experiment 2:
CREB

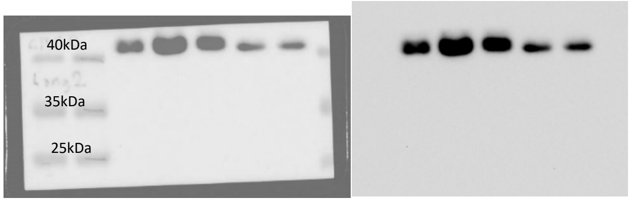

Tubulin

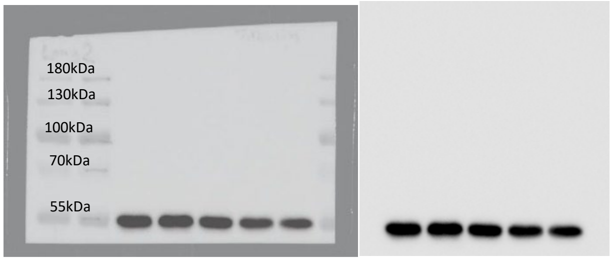

Experiment 3:
Tubulin (upper band) and CREB (lower band)

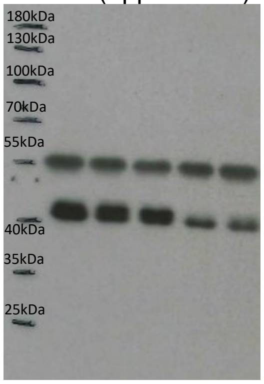

Experiment 4:
CREB

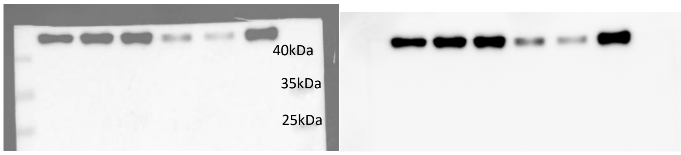

Tubulin

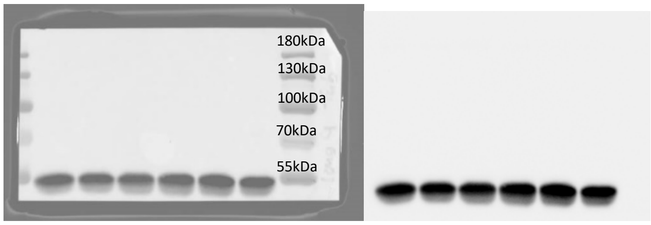

Fig. 4G: ERK1/2, long protocol (1 h + 1 h), n=4
Sample order always from left to right.
1 = untreated Ctrl
2 = 20 min Bic
3 = 10 min Bic + 10 min Bic/TTX
4 = 10 min Bic + 10 min Bic/TTX/NMDA
5 = 10 min Bic + 10 min Bic/NMDA

Experiment 1:
ERK1/2

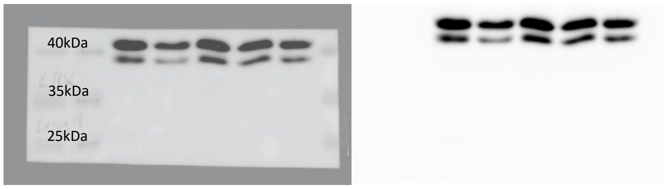

Tubulin

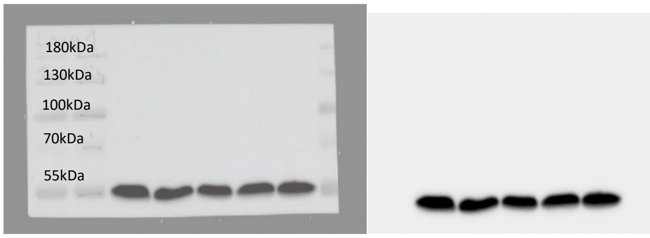

Experiment 2:
ERK1/2

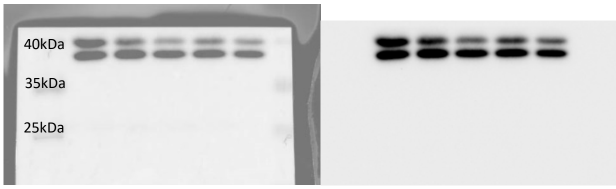

Tubulin

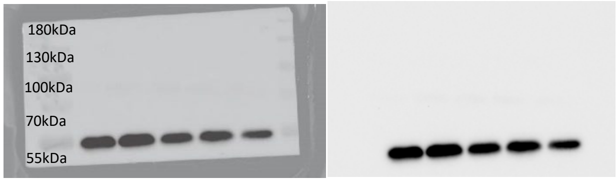

Experiment 3:
ERK1/2

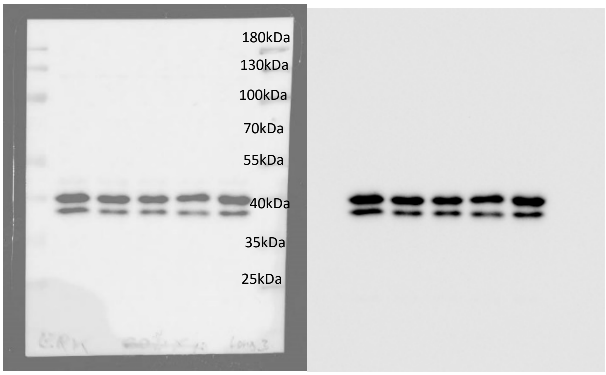

Tubulin

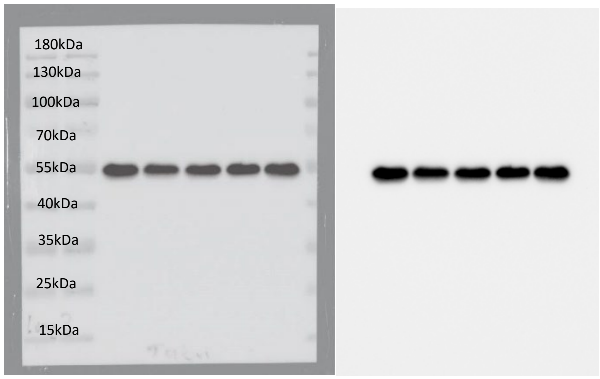

Experiment 4:
ERK1/2

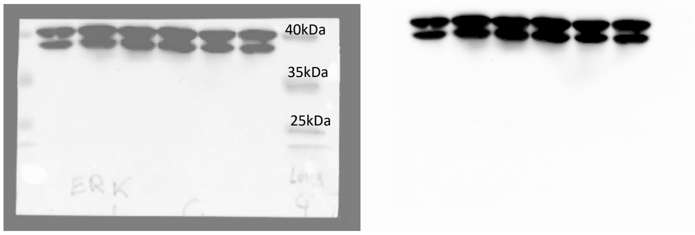

Tubulin

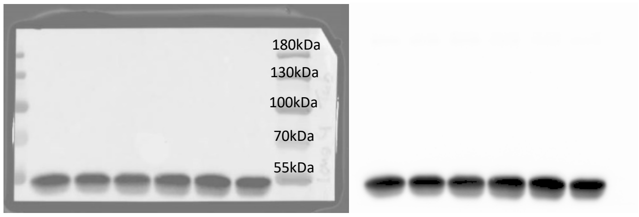

Supplement: Supplementary file 1 — Supplementary Information [file 42003_2025_9074_MOESM1_ESM.pdf]
