# Supplementary material for: Inhba, Homer1 and Bdnf are major targets of transcriptomic dysregulation by neurodegenerative disease-associated excitotoxic NMDA receptor signaling
Source: Commun Biol. 2025 Dec 3;8:1743. doi: 10.1038/s42003-025-09074-9 (PMC12678830; doi:10.1038/s42003-025-09074-9)
Supplement: Supplementary file 2 — Description of Additional Supplementary Files [file 42003_2025_9074_MOESM2_ESM.pdf]

Description of Additional Supplementary Files

File name: Supplementary Data 1
Description: “Combined, comprehensive analyses results of the RNA sequencing experiment conducted in this study”

File name: Supplementary Data 2
Description: **“**Source data of results shown in Fig. 1C and Figs. 4 – 6.”
