# Supplementary material for: Inhba, Homer1 and Bdnf are major targets of transcriptomic dysregulation by neurodegenerative disease-associated excitotoxic NMDA receptor signaling
Source: Commun Biol. 2025 Dec 3;8:1743. doi: 10.1038/s42003-025-09074-9 (PMC12678830; doi:10.1038/s42003-025-09074-9)
Supplement: Supplementary file 5 — Reporting Summary [file 42003_2025_9074_MOESM5_ESM.pdf]

nature portfolio

Corresponding author(s): Hilmar Bading

Last updated by author(s): Jan 14, 2025

Reporting Summary

Nature Portfolio wishes to improve the reproducibility of the work that we publish. This form provides structure for consistency and transparency in reporting. For further information on Nature Portfolio policies, see our Editorial Policies and the Editorial Policy Checklist.

Statistics

For all statistical analyses, confirm that the following items are present in the figure legend, table legend, main text, or Methods section.

n/a	Confirmed	
☐	☒	The exact sample size (*n*) for each experimental group/condition, given as a discrete number and unit of measurement
☐	☒	A statement on whether measurements were taken from distinct samples or whether the same sample was measured repeatedly
☐	☒	The statistical test(s) used AND whether they are one- or two-sided<br>*Only common tests should be described solely by name; describe more complex techniques in the Methods section.*
☒	☐	A description of all covariates tested
☐	☒	A description of any assumptions or corrections, such as tests of normality and adjustment for multiple comparisons
☐	☒	A full description of the statistical parameters including central tendency (e.g. means) or other basic estimates (e.g. regression coefficient) AND variation (e.g. standard deviation) or associated estimates of uncertainty (e.g. confidence intervals)
☐	☒	For null hypothesis testing, the test statistic (e.g. *F*, *t*, *r*) with confidence intervals, effect sizes, degrees of freedom and *P* value noted<br>*Give P values as exact values whenever suitable.*
☒	☐	For Bayesian analysis, information on the choice of priors and Markov chain Monte Carlo settings
☒	☐	For hierarchical and complex designs, identification of the appropriate level for tests and full reporting of outcomes
☒	☐	Estimates of effect sizes (e.g. Cohen's *d*, Pearson's *r*), indicating how they were calculated

Our web collection on statistics for biologists contains articles on many of the points above.

Software and code

Policy information about availability of computer code

Data collection	Illumina bcl2fastq2 Conversion Software v2.20 for transferring base calls to reads was used for data collection.
Data analysis	Open source software used:<br>Read mapping: STAR (2.5.4b) (Dobin et al., 2013)<br>Read counting: FeatureCounts (1.6.5) (Liao et al., 2014)<br>RNA-seq expression analysis: R (3.6.3) (http://www.R-project.org/)<br>Differentially expressed genes: DESeq2 R package (1.26.0) (Love et al., 2014)<br>tSNE analysis: Rtsne R package (0.15) (https://github.com/jkrijthe/Rtsne)<br>clusterProfiler R package (v4.10.0)

For manuscripts utilizing custom algorithms or software that are central to the research but not yet described in published literature, software must be made available to editors and reviewers. We strongly encourage code deposition in a community repository (e.g. GitHub). See the Nature Portfolio guidelines for submitting code & software for further information.

Data

Policy information about availability of data

All manuscripts must include a data availability statement. This statement should provide the following information, where applicable:

- Accession codes, unique identifiers, or web links for publicly available datasets
- A description of any restrictions on data availability
- For clinical datasets or third party data, please ensure that the statement adheres to our policy

RNA-seq data of this publication have been deposited in NCBI's Gene Expression Omnibus (GSE184681).

Research involving human participants, their data, or biological material

Policy information about studies with human participants or human data. See also policy information about sex, gender (identity/presentation), and sexual orientation and race, ethnicity and racism.

Reporting on sex and gender	*Use the terms sex (biological attribute) and gender (shaped by social and cultural circumstances) carefully in order to avoid confusing both terms. Indicate if findings apply to only one sex or gender; describe whether sex and gender were considered in study design; whether sex and/or gender was determined based on self-reporting or assigned and methods used. Provide in the source data disaggregated sex and gender data, where this information has been collected, and if consent has been obtained for sharing of individual-level data; provide overall numbers in this Reporting Summary. Please state if this information has not been collected. Report sex- and gender-based analyses where performed, justify reasons for lack of sex- and gender-based analysis.*
Reporting on race, ethnicity, or other socially relevant groupings	*Please specify the socially constructed or socially relevant categorization variable(s) used in your manuscript and explain why they were used. Please note that such variables should not be used as proxies for other socially constructed/relevant variables (for example, race or ethnicity should not be used as a proxy for socioeconomic status). Provide clear definitions of the relevant terms used, how they were provided (by the participants/respondents, the researchers, or third parties), and the method(s) used to classify people into the different categories (e.g. self-report, census or administrative data, social media data, etc.) Please provide details about how you controlled for confounding variables in your analyses.*
Population characteristics	*Describe the covariate-relevant population characteristics of the human research participants (e.g. age, genotypic information, past and current diagnosis and treatment categories). If you filled out the behavioural & social sciences study design questions and have nothing to add here, write "See above."*
Recruitment	*Describe how participants were recruited. Outline any potential self-selection bias or other biases that may be present and how these are likely to impact results.*
Ethics oversight	*Identify the organization(s) that approved the study protocol.*

Note that full information on the approval of the study protocol must also be provided in the manuscript.

Field-specific reporting

Please select the one below that is the best fit for your research. If you are not sure, read the appropriate sections before making your selection.

☒ Life sciences ☐ Behavioural & social sciences ☐ Ecological, evolutionary & environmental sciences

For a reference copy of the document with all sections, see nature.com/documents/nr-reporting-summary-flat.pdf

Life sciences study design

All studies must disclose on these points even when the disclosure is negative.

Sample size	Sample size of in vitro experiments was determined from experience of our previous CREB shut-off study (Hardingham et al., 2002). Animal number was predetermined with a power calculation (80% power) based on exploratory RTqPCR data from R6/2 Huntington´s disease model mice.
Data exclusions	Two samples from untreated zQ175 were excluded from RTqPCR data since they were identified as outliers with the robust regression and outlier removal test (ROUT, Q=0.5%). They were excluded for all analyzed genes and regions. No other data was excluded.
Replication	In vitro RTqPCR data was gained from eight independent preparations of primary neurons, in vitro sequencing data from five independent preparations and in vivo RTqPCR data from two different litters.
Randomization	Allocation of treatment conditions on cell culture plates was randomized. Assignment of treatment groups for WT and zQ175 mice was randomized.
Blinding	The scientist performing brain dissections for in vivo samples was blinded to the genotype of the animals. All other applied quantification

Blinding | methods were automated and independent of the scientist (cell death analysis, RTqPCR quantification, transcriptome analysis).

Reporting for specific materials, systems and methods

We require information from authors about some types of materials, experimental systems and methods used in many studies. Here, indicate whether each material, system or method listed is relevant to your study. If you are not sure if a list item applies to your research, read the appropriate section before selecting a response.

Materials & experimental systems

n/a	Involved in the study
☐	☒ Antibodies
☒	☐ Eukaryotic cell lines
☒	☐ Palaeontology and archaeology
☐	☒ Animals and other organisms
☒	☐ Clinical data
☒	☐ Dual use research of concern
☒	☐ Plants

Methods

n/a	Involved in the study
☒	☐ ChIP-seq
☒	☐ Flow cytometry
☒	☐ MRI-based neuroimaging

Antibodies

Antibodies used

The following primary antibodies were used: anti-phospho-CREB (Ser133) (Millipore, #06-519); anti-CREB (Cell Signaling, #9197); anti-phospho-Elk1 (Ser383) (Santa Cruz, #sc-8406); anti-phospho-ERK1/2 (Thr 202/ Tyr 204) (Cell Signaling, #9106); anti-ERK1/2 (Cell Signaling, #9102); anti-total phospho-Ser/Thr (ECM Biosciences, #PP2551); anti-alpha-tubulin (Sigma, #T9026). The following respective secondary antibodies were used: anti-mouse IgG-HRP (Jackson ImmunoResearch, #115-035-003); anti-rabbit IgG-HRP (Jackson ImmunoResearch, #115-035-144).

Validation

Millipore, #06-519 is highly published and validated in EMSA, IHC,IHC(P), IP, WB to detect phospho-CREB (Ser133) also known as active transcription factor CREB, cAMP responsive element binding protein 1. Evaluated by Western Blotting in Forskolin treated BALB/3T3 clone A31 cell lysate. Western Blotting Analysis: A 1:1,000 dilution of this antibody detected phospho CREB (Ser133) in 10 µg of Forskolin treated BALB/3T3 clone A31 cell lysate.
Cell Signaling, #9197 has 1184 product citations. It detects endogenous levels of total CREB-1 protein. The antibody does not cross-react with other ATF/CREB family members.
Santa Cruz, #sc-8406 is a mouse monoclonal IgG1 κ p-Elk-1 Antibody, cited in 88 publications that is recommended for detection of p-Elk-1 phosphorylated at Ser 383 of mouse, rat and human origin by WB, IP, IF, IHC(P) and ELISA.
Cell Signaling, #9106 has 1956 product citations. It detects endogenous levels of p44 and p42 MAP Kinase (Erk1 and Erk2) when dually phosphorylated at Thr202 and Tyr204 of Erk1 (Thr185 and Tyr187 of Erk2), but not singly phosphorylated at Thr202 or Tyr204. This antibody does not cross-react with the corresponding phosphorylated residues of either SAPK/JNK or p38 MAP kinase.
Cell Signaling, #9102 has 7994 product citations. It detects endogenous levels of total p44/42 MAP kinase (Erk1/Erk2) protein. In some cell types, this antibody recognizes p44 MAPK more readily than p42 MAPK. The antibody does not recognize either JNK/SAPK or p38 MAP kinase.
ECM Biosciences, #PP2551 has 11 product citations. It was cross-adsorbed to unphosphorylated peptide then affinity purified using a mix of phosphoserine and phosphothreonine peptides (without carrier). The antibody detects many serine or threonine phosphorylated proteins by western blot, immunocytochemistry, and ELISA.
Sigma, #T9026 is a Merck enhanced validation mouse monoclonal anti-α-Tubulin antibody.

Animals and other research organisms

Policy information about studies involving animals; ARRIVE guidelines recommended for reporting animal research, and Sex and Gender in Research

Laboratory animals

We used 24-26-week-old C57B/l6J background zQ175 mice and their WT littermates. zQ175 mice have a chimeric human/mouse exon 1 of the huntingtin gene, carrying the expanded CAG repeat region and the human polyproline region of ˜188 CAG repeats.

Wild animals

The study did not involve wild animals.

Reporting on sex

37 mice were used in this study, each analyzed group contained a mix of both genders (in total 15 female; 22 male).

Field-collected samples

The study did not involve samples collected from the field.

Ethics oversight

This study was approved by the responsible animal care committee (Regierungspräsidium Karlsruhe, Germany, approval G-102/16). The authors confirm that all experiments were carried out in accordance with German guidelines for the care and use of laboratory animals and with the European Community Council Directive 86/609/EEC.

Note that full information on the approval of the study protocol must also be provided in the manuscript.

Plants

Seed stocks	*Report on the source of all seed stocks or other plant material used. If applicable, state the seed stock centre and catalogue number. If plant specimens were collected from the field, describe the collection location, date and sampling procedures.*
Novel plant genotypes	*Describe the methods by which all novel plant genotypes were produced. This includes those generated by transgenic approaches, gene editing, chemical/radiation-based mutagenesis and hybridization. For transgenic lines, describe the transformation method, the number of independent lines analyzed and the generation upon which experiments were performed. For gene-edited lines, describe the editor used, the endogenous sequence targeted for editing, the targeting guide RNA sequence (if applicable) and how the editor was applied.*
Authentication	*Describe any authentication procedures for each seed stock used or novel genotype generated. Describe any experiments used to assess the effect of a mutation and, where applicable, how potential secondary effects (e.g. second site T-DNA insertions, mosiacism, off-target gene editing) were examined.*
